# Integrative single-cell multiomics analyses dissect molecular signatures of intratumoral heterogeneities and differentiation states of human gastric cancer

**DOI:** 10.1093/nsr/nwad094

**Published:** 2023-04-11

**Authors:** Shuhui Bian, Yicheng Wang, Yuan Zhou, Wendong Wang, Limei Guo, Lu Wen, Wei Fu, Xin Zhou, Fuchou Tang

**Affiliations:** Biomedical Pioneering Innovation Center, School of Life Sciences, Department of General Surgery, Third Hospital, Peking University, Beijing 100871, China; State Key Laboratory of Reproductive Medicine and Offspring Health, Nanjing Medical University, Nanjing 211166, China; Collaborative Innovation Center for Cancer Personalized Medicine, Nanjing Medical University, Nanjing 211166, China; Biomedical Pioneering Innovation Center, School of Life Sciences, Department of General Surgery, Third Hospital, Peking University, Beijing 100871, China; Beijing Advanced Innovation Center for Genomics (ICG), Ministry of Education Key Laboratory of Cell Proliferation and Differentiation, Beijing 100871, China; Biomedical Pioneering Innovation Center, School of Life Sciences, Department of General Surgery, Third Hospital, Peking University, Beijing 100871, China; Beijing Advanced Innovation Center for Genomics (ICG), Ministry of Education Key Laboratory of Cell Proliferation and Differentiation, Beijing 100871, China; Biomedical Pioneering Innovation Center, School of Life Sciences, Department of General Surgery, Third Hospital, Peking University, Beijing 100871, China; Department of Pathology, Peking University Third Hospital, School of Basic Medical Science, Peking University Health Science Center, Beijing 100191, China; Biomedical Pioneering Innovation Center, School of Life Sciences, Department of General Surgery, Third Hospital, Peking University, Beijing 100871, China; Beijing Advanced Innovation Center for Genomics (ICG), Ministry of Education Key Laboratory of Cell Proliferation and Differentiation, Beijing 100871, China; Biomedical Pioneering Innovation Center, School of Life Sciences, Department of General Surgery, Third Hospital, Peking University, Beijing 100871, China; Peking University Third Hospital Cancer Center, Beijing 100191, China; Biomedical Pioneering Innovation Center, School of Life Sciences, Department of General Surgery, Third Hospital, Peking University, Beijing 100871, China; Peking University Third Hospital Cancer Center, Beijing 100191, China; Biomedical Pioneering Innovation Center, School of Life Sciences, Department of General Surgery, Third Hospital, Peking University, Beijing 100871, China; Beijing Advanced Innovation Center for Genomics (ICG), Ministry of Education Key Laboratory of Cell Proliferation and Differentiation, Beijing 100871, China; Peking-Tsinghua Center for Life Sciences, Academy for Advanced Interdisciplinary Studies, Peking University, Beijing 100871, China

**Keywords:** gastric cancer, single-cell multiomics analysis, intratumoral heterogeneity, tumor differentiation

## Abstract

Human gastric cancer is a highly lethal disease, but the underlying multiomic molecular signatures remain largely unclear. Here, we performed multi-regional sampling, parallel single-cell multiomics sequencing and integrated analyses of human gastric cancer. We identified common transcriptomic alterations of gastric cancer cells, such as aberrant down-regulation of genes associated with normal stomach function and up-regulation of *KRT7, PI3, S100A4*, etc. Surprisingly, aberrant and prevalent up-regulation of genes highly expressed in normal colorectal epithelial cells were also identified in cancer cells, which may be partially regulated by promoter chromatin accessibility and DNA methylation levels. We revealed the single-cell DNA methylome landscape of gastric cancer, and identified candidate DNA methylation biomarkers, such as hypermethylated promoters of *TMEM240* and *HAGLROS*, and hypomethylated promoters of *TRPM2-AS* and *HRH1.* Additionally, the relationships between genetic lineages, DNA methylation and transcriptomic clusters were systematically revealed at single-cell level. We showed that DNA methylation heterogeneities were mainly among different genetic lineages of cancer cells. Moreover, we found that DNA methylation levels of cancer cells with poorer differentiation states tend to be higher than those of cancer cells with better differentiation states in the primary tumor within the same patient, although still lower than in normal gastric epithelial cells. Cancer cells with poorer differentiation states also prevalently down-regulated *MUC1* expression and immune-related pathways, and had poor infiltration of CD8^+^ T cells. Our study dissected the molecular signatures of intratumoral heterogeneities and differentiation states of human gastric cancer using integrative single-cell multiomics analyses.

## INTRODUCTION

Gastric cancer (GC) is a highly heterogeneous disease with high morbidity and mortality worldwide [1]. The tumor heterogeneity of GC involves histopathological, genetic, epigenetic and transcriptomic aspects [2]. The Lauren's classification classified GC into intestinal and diffuse types [3], and tumors with components from two types were grouped as mixed type. The WHO classification, which is another histopathological classification system, divided GC into more detailed histopathological subtypes and differentiation states, such as papillary/tubular/mucinous adenocarcinoma (ADC), poorly cohesive carcinoma, signet-ring cell carcinoma, neuroendocrine carcinoma (NEC), hepatoid adenocarcinoma (HAS) and mixed carcinoma [4].

Although different histopathological tumors have significant differences in clinical behavior, the histopathology-based classification still has little clinical utility in guiding therapies for GC patients currently. One of the core reasons is our limited understanding of the molecular basis and oncogenic targets of different histopathological subtypes. Further investigation into the molecular mechanisms of GC, and the identification of specific molecular mechanisms of different subtypes, are of urgent priority.

Intratumoral heterogeneity (ITH) has posed severe challenges to accurate diagnosis and personalized therapy, and can lead to drug resistance, cancer recurrence, metastasis and poor patient outcomes [5,6]. Among the diverse histopathology-based subtypes, the mixed carcinoma of GC contains more than one histopathological components and is a representative example of drastic ITH, such as mixed adeno-neuroendocrine carcinoma (MANEC). The mixed GCs lack specific and efficient treatment strategies, have a worse prognosis than GC with only ADC, and are prone to metastasis [7].

There have been several molecular studies of GC. The large-scale molecular profiling studies, such as The Cancer Genome Atlas (TCGA) and the Asian Cancer Research Group, have identified molecular subtypes of GC [2,8], which provide important supplements to histopathological classification. However, these studies are based on bulk sequencing technologies, and have limited ability to capture ITH within each tumor. Additionally, single-cell sequencing provides powerful tools to characterize ITH, and several single-cell RNA-seq studies have revealed the ITH of GCs recently [9–13]. For example, Zhang *et al.* identified a panel of differentiation-related genes of gastric ADC [13]. However, they mainly focused on the transcriptome of gastric ADC, and other omics and non-ADC subtypes and mixed-type GC were largely unexplored. The relationships among the genome, DNA methylome and transcriptome of GC have still not been systematically revealed at single-cell level. Hence, it is urgently needed to reveal the heterogeneity of GC more thoroughly and at single-cell multiomics levels, and provide novel insights for clinical treatment strategies.

In this study, we aimed to dissect multiomics ITH at single-cell resolution, and identify the specific molecular basis of different differentiation states of GC. We performed multi-regional sampling, single-cell multiomics sequencing (which can profile genetic, epigenetic and transcriptomic features simultaneously in the same individual cell) and integrated side-by-side histopathological analyses of human GC, providing a deeper understanding of the ITH and the underlying molecular basis of GC.

## RESULTS

### Multi-regional sampling and single-cell multiomics sequencing of human gastric cancer

**Figure 1.**
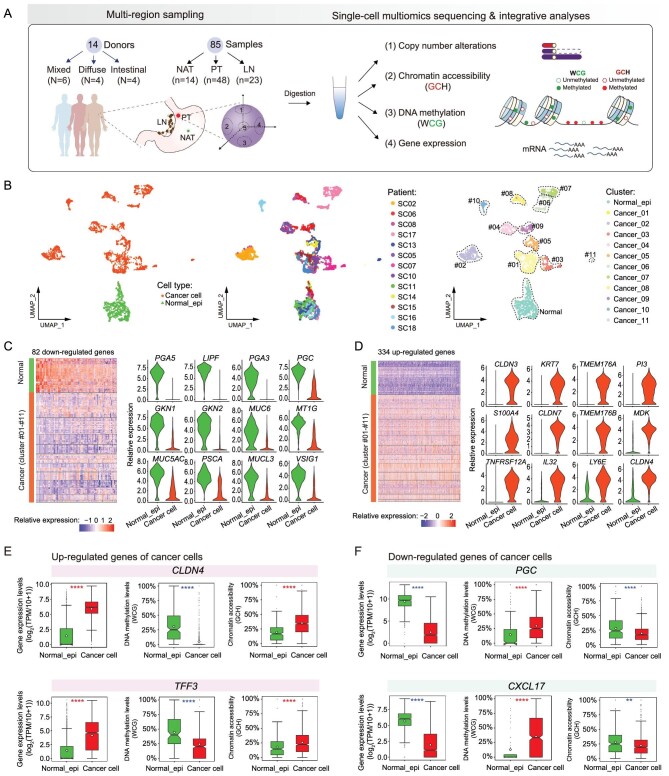
Common transcriptomic alterations of cancer cells compared with normal epithelial cells. (A) Schematic illustration of the study workflow. WCG, W denotes A or T. GCH, H denotes A, C or T. (B) The UMAP of all cancer cells and normal_epi_stomach based on scTrio-seq3 transcriptome data. (C) and (D) Highly expressed DEGs of normal_epi_stomach (C) and cancer cells (D). (E) and (F) The gene expression levels, DNA methylation levels and chromatin accessibility levels of related promoter regions of representative genes. The white diamonds represent the mean value of each group. Wilcoxon rank-sum test, ****P-value < 0.0001, ***P-value < 0.001, **P-value < 0.01, *P-value < 0.05.

Our optimized single-cell multiomics sequencing method (scTrio-seq3), enables simultaneous profiling of somatic copy number alterations (SCNAs), chromatin accessibility, DNA methylation and RNA expression in the same individual cell [14]. In this study, we performed scTrio-seq3 analysis of 14 patients (Figs 1A and S1A; Table S1). According to the Lauren's classification, the patients can be divided into four intestinal-type, four diffuse-type and six mixed-type patients. Paired normal adjacent tissues (NATs), primary tumors (PTs) and lymph node metastases (LNs) were sampled (for 10/14 patients). For each tumor, multi-regional sampling was performed (for 13/14 patients). Side-by-side histopathological analyses of each sampling region were performed by at least two pathologists.

To further dissect the ITH of GC, the tumor samples were further classified as ADC (poorly differentiated, moderately differentiated or well differentiated), NEC or HAS based on the histopathological features according to the WHO classification system. Cancer cells with different differentiation states can coexist within the same patient, and the GCs with mixed differentiation states (mGCs) (SC02, SC06, SC07, SC08, SC13 and SC17) provided a good opportunity to explore specific features of different differentiation states.

### Common transcriptomic alterations of cancer cells compared with normal epithelial cells

With high-quality single-cell transcriptome data, we performed a uniform manifold approximation and projection (UMAP) analysis of cancer cells and normal gastric epithelial cells (normal_epi_stomach). As a result, the normal_epi_stomach from all sampled patients clustered together, whereas cancer cells showed strong inter-patient heterogeneities (Fig. 1B). As shared gene expression alterations of GC are important for the understanding of GC tumorigenesis, we identified differentially expressed genes (DEGs) between the cancer cells and normal_epi_stomach of all sampled patients. As a result, 334 genes were up-regulated and 82 genes were down-regulated in most cancer cells of all patients under stringent cut-off (Supplementary Methods) (Fig. 1C and D; Table S2).

Interestingly, we found that several highly expressed genes of normal colorectal epithelial cells (normal_epi_colon) were among the up-regulated DEGs of GC cells (Fig. 1D). To further explore this phenomenon, we compared the DEGs between normal_epi_stomach and normal_epi_colon using single-cell data of normal_epi_stomach in this study and single-cell data of normal_epi_colon from a study previously published by our group [15] using stringent cut-off (Supplementary Methods), and obtained 158 normal_epi_stomach highly expressed genes and 111 normal_epi_colon highly expressed genes. We found that 23.4% (26/111) of normal_epi_colon highly expressed genes were aberrantly up-regulated in GC cells (Fig. S1B–D), such as goblet cell marker gene *TFF3*, claudin family genes (*CLDN3, CLDN4* and *CLDN7*), transmembrane protein genes (*TMEM176A* and *TMEM176B*) and mucin gene (*MUC13*), which were shared by all GC patients in our study (Fig. S1C and D) and were validated in Human Protein Atlas data sets (https://www.proteinatlas.org/) (Fig. S1E). The SC14 NAT also highly expressed some normal_epi_colon highly expressed genes, which was consistent with its apparent intestinal metaplasia (Fig. S1F). Our data show that up-regulation of many normal_epi_colon highly expressed genes is a prevalent signature of the cancer cells of GC.

Additionally, we found that 51.3% (81/158) of the normal_epi_stomach highly expressed genes were significantly down-regulated in GC cells (Figs 1C and S1G), such as the pepsinogen genes (*PGA3, PGA5* and *PGC*), lipase gene (*LIPF*), gastrokine genes (*GKN1* and *GKN2*) and some mucin genes (*MUC1, MUC5AC* and *MUC6*), which indicated the general loss of normal stomach functions in GC cells. Together, these results suggest the possibility that during the tumorigenesis process of GC, the epithelial cells lose the normal physiological functions of gastric epithelial cells, become more flexible and gain higher plasticity, which even permits them to start to express many feature genes of the colon epithelial cells.

Furthermore, we searched for the DEGs with altered epigenetics features (Table S2), and found that increased chromatin accessibility and/or decreased DNA methylation levels in promoter regions may explain the up-regulation of some normal_epi_colon highly expressed genes in cancer cells, such as *CLDN3, CLDN4, TFF3* and *MISP* (Figs 1E and S1H; Table S2). Additionally, decreased chromatin accessibility and/or increased DNA methylation levels in promoter regions may explain the down-regulation of some normal_epi_stomach highly expressed genes, such as *PGC* and *CXCL17* (Fig. 1F; Table S2). These data show that promoter chromatin accessibility and DNA methylation levels may participate in the aberrant gain of normal_epi_colon signatures and loss of normal_epi_stomach signatures in GC cells, providing important clues with regard to their epigenetic regulation during the tumorigenesis process of GC.

### Single-cell DNA methylation map of cancer cells of gastric cancer

Next, we explored the DNA methylation changes of cancer cells. The cancer cells had undergone strong DNA demethylation, especially on repeat elements (such as long interspersed element, long terminal repeat and satellite), with both inter-patient and intra-patient heterogeneities (Fig. S2A and B). We noticed that compared with our previous study in colorectal cancer [16], the DNA demethylation of GC was more drastic. For example, the median DNA methylation levels in all cancer cells of the whole genome, L1 and satellite were 55%, 44% and 37%, respectively (Fig. S2A–C).

**Figure 2.**
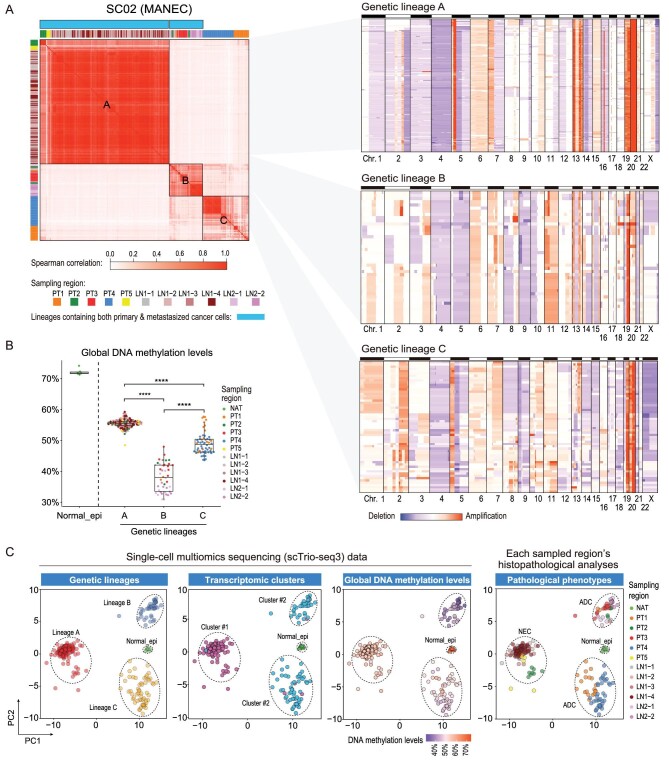
Genetic lineages, DNA methylation levels and transcriptomic clusters of mGC patient SC02. (A) The left heatmap shows the Spearman correlations of SCNA profiles of SC02. The right three heatmaps show the SCNA profiles of each genetic lineage of SC02. The cells are ordered according to the results of the hierarchical clustering of SCNA profiles. (B) The global DNA methylation levels of each genetic lineage of SC02. Wilcoxon rank-sum test, ****P-value < 0.0001. (C) The PCA projection of SC02 SCNA profiles based on scTrio-seq3 DNA data.

As DNA methylation is considered to play a critical role in silencing transposons and maintaining genome stability [17], we explored the effect of such drastic genome-wide DNA demethylation. Long interspersed element-1 (LINE-1, L1) is a transposable element with retrotransposition potential in the human genome, and is mainly silenced by DNA methylation in normal contexts and may be reactivated in cancer [17,18]. We observed drastically decreased DNA methylation levels of L1 in cancer cells (Fig. S2C). Furthermore, we performed immunohistochemical (IHC) staining of L1 ORF1p protein in GC tissues (Fig. S2D) and observed its abnormal expression in cancer cells; in contrast, ORF1p was not detected in either normal_epi_stomach or stromal cells of the stomach. The result indicated that the retrotransposon L1 may aberrantly reactivate in GC and destruct genome integrity, which is consistent with the highly disordered SCNA profiles of GCs (Figs 2A and S4).

Promoter DNA methylation and chromatin accessibility profiles can distinguish normal_epi_stomach and cancer cells (Fig. S3A and B). We found 455 significantly hypermethylated differentially methylated gene promoters (DMPs) in cancer cells on average, and 116 hypomethylated DMPs on average among 14 patients (Fig. S3C; Table S3). Moreover, several DMPs were shared by >50% of the patients (Fig. S3D), indicating the consistent tendency of DNA methylation alterations to occur, and providing candidate DNA methylation biomarkers of human GC. For example, hypermethylated promoters of transmembrane protein 240 (*TMEM240*) and HAGLR opposite strand lncRNA (*HAGLROS*) were observed in the cancer cells of most patients (11/14), while hypomethylated promoters of *TRPM2-AS* and histamine receptor H1 (*HRH1*) were observed in the cancer cells of 10/14 patients.

### Relationships among genetic lineages, DNA methylation and transcriptomic clusters of mGC

To delineate the genetic lineages of mGC, we performed single-cell SCNA analyses for each mGC patient. The individual cancer cells showed a high frequency of copy number gains and losses across the genome and displayed complex SCNA patterns, illustrating high chromosome instability (Figs 2A and S4). We identified genetic lineages of individual cancer cells (Figs 2A and S4) based on unsupervised hierarchical clustering of the single-cell SCNA profiles, and found that each of the six mGC patients had multiple lineages of cancer cells. Within each patient, the correlations of SCNA profiles between different lineages were relatively low (Figs 2A and S4), indicating high inter-lineage heterogeneities.

Our single-cell multiomic sequencing data help to explore the epigenetic features of cancer cells in each genetic lineage. We found that global DNA methylation heterogeneities mainly existed among different genetic lineages, and the global DNA methylation characteristics were relatively consistent within each genetic lineage of cancer cells (Figs 2B and S5A). Unsupervised hierarchical clustering of genome-wide DNA methylation profiles can also generate cancer cell clusters highly consistent with their genetic lineages (Fig. S5B). The results suggested that the global DNA methylation characteristics were maintained within the same genetic lineage of cancer cells during tumorigenesis and progression. Additionally, we noticed the genetic lineages containing both primary cancer cells and metastasized cancer cells in 5/6 mGC patients, which provided an opportunity for tracing the molecular changes between the PT and LN of the same genetic lineage. We observed that multiple trends of global DNA methylation changes between PT and LN within the same genetic lineage coexist (Fig. S5C). However, only SC02 lineage B and SC06 lineage B showed relatively apparent changes (with differences of median values >5%), while the others’ changes were quite mild.

We performed UMAP analyses of transcriptome data for each mGC patient, and explored the relationships between genetic lineages, transcriptomic clusters and the spatial positions of the tumors in mGC patients (Figs 2C and S6). We noticed that one genetic lineage of cancer cells can correspond to multiple transcriptomic clusters, and different genetic lineages can correspond to the same transcriptomic cluster. Additionally, cancer cells from different spatial positions of the same tumor showed drastic ITH, and cancer cells from the same sampling position could also belong to different genetic lineages or transcriptomic clusters, manifesting high spatial heterogeneities of mGC.

### Identifying the differentiation states of cancer cells in mGC

**Figure 3.**
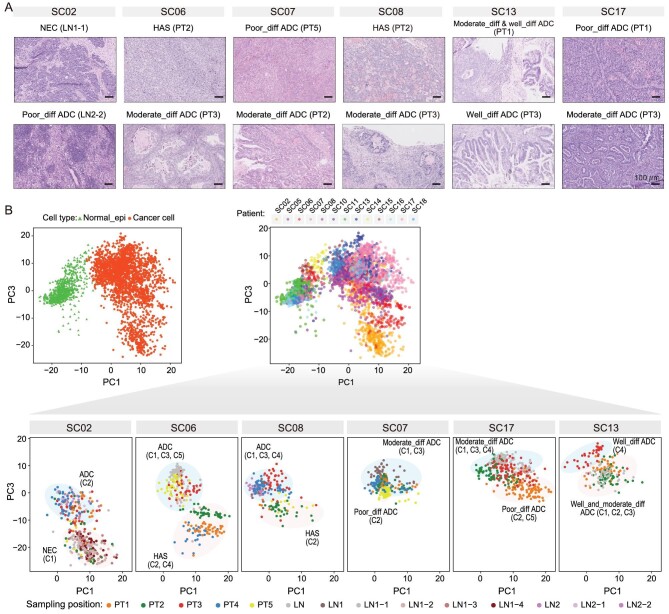
Identification of the differentiation states of cancer cells in mGC. (A) H&E staining of tumor tissues from patients with mGC. Diff, differentiation. Scale bars, 100 μm. (B) The PCA projection using the transcriptome data from single-cell multiomics sequencing. The transcriptome cluster IDs of six mGC patients are labeled.

To identify the differentiation states of cancer cells in mGC patients, we further explored the DEGs among transcriptomic clusters for each mGC patient (Fig. S7; Table S4). We found that our single-cell gene expression data and multi-regional histopathological analyses results were, in general, consistent with each other (Figs 3 and S7). For example, the cancer cells of SC02 (a MANEC patient) were divided into two major transcriptomic clusters (Fig. S7). Cluster #1 highly expressed some classical neuroendocrine marker genes (such as chromogranin B gene *CHGB* and secretogranin-3 gene *SCG3*), and the sampling regions of cluster #1 (PT5, LN1-1, LN1-2, LN1-3 and LN1-4) were classified as as NEC according to the histopathological features. In contrast, cluster #2 had higher expression levels of some members of the mucin family, such as *MUC1, MUC5B* and *MUC6*, than cluster #1, and the sampling regions of cluster #2 (PT1, PT3, PT4, LN2-1 and LN2-2) were classified as ADC according to the histopathological features. Additionally, the cancer cells from PT2 of SC02 belonged to both transcriptomic cluster #1 and #2, although the histopathological results mainly supported NEC, probably due to the spatial heterogeneities between different parts in the same sampling region.

For SC06 (an mGC patient with HAS and ADC), cluster #2 highly expressed two classical marker genes of HAS (alpha-fetoprotein gene (*AFP*) and glypican 3 (*GPC3*)), and the sampling regions of cluster #2 (PT1 and PT4) were classified as HAS according to the histopathological features. Cluster #3 and cluster #5 had higher expression levels of *MUCL3, MUC5AC, MUC5B* and *MUC17* than other clusters, and the sampling regions of cluster #2 (PT3 and PT5) were classified as ADC according to the histopathological features. The PT2 (cluster #4) of SC06 was classified as HAS according to histopathological features, which did not express *AFP* but expressed *GPC3*, suggesting that gene expression heterogeneities exist between tumors with similar histopathological features. In another mGC patient (SC08), elevated serum AFP levels were detected. The transcriptomic cluster #2 of SC08 did not express *AFP* but highly expressed *GPC3* and multiple genes that were highly expressed in normal liver and liver cancer, such as apolipoprotein genes (*APOA1, APOA2* and *APOB*), transferrin gene (*TF*), fibrinogen genes (*FGG* and *FGA*) and coagulation factor II gene (*F2*), and the sampling regions of cluster #2 (PT1, PT2 and PT5) were classified as HAS according to the histopathological features. Moreover, in the UMAP consisting of cells of all patients, cancer cells of HAS from both SC06 and SC08 clustered together in the cluster Cancer_08 (Fig. 1B). The PT3 (cluster #4) and PT4 (cluster #3) of SC08 were classified as ADC, and highly expressed *MUC1* and *CLDN18* (Table S5). Hence, we can classify major differentiation states of ADC and non-ADC based on single-cell gene expression features and histopathological characteristics collectively.

Although ADC can be divided into well-differentiated, moderately differentiated or poorly differentiated subtypes based on histopathological features, identifying the differentiation states of ADC for individual cancer cells is really challenging. There are two major reasons for this: (i) there are currently no specific and general marker genes for different differentiation states of ADC available due to drastic inter-patient heterogeneity; (ii) although histopathological features provide strong evidence when identifying the diverse differentiation states of ADC, they should not be the only evidence because drastic spatial heterogeneities may exist within the same sampling region in mGC patients. Hence, we aimed to further identify the differentiation states of cancer cells (especially for ADC), taking advantage of our multi-regional sampling, histopathological analysis and single-cell multiomics sequencing. Since the UMAP of cells from all patients displayed relatively strong inter-patient heterogeneities, we tried to perform principal component analysis (PCA) of normal_epi_stomach and cancer cells of all patients (Fig. 3B). We found that the PC1 axis can clearly separate normal_epi_stomach cells from cancer cells. Moreover, the PC3 axis can divide transcriptomic clusters into two major differentiation states within each mGC patient. Intriguingly, we found that cancer cells with different differentiation states of ADC (based on histopathological features) were also separated along the PC3 axis within each mGC patient (SC07, SC13 and SC17); cancer cells with better differentiation states tend to have PC3 coordinates with higher values, and those with poorer differentiation states tend to be located at PC3 coordinates with lower values, within each mGC patient. Hence, we can identify the differentiation states for cancer cells of six mGC patients by integration of single-cell multiomics and multi-regional histopathological analyses.

### Identifying molecular features of cancer cells with different differentiation states

After identification of differentiation states in each mGC patient, our single-cell multiomics sequencing data provided a good chance to further dissect the molecular features of cancer cells with different differentiation states.

**Figure 4.**
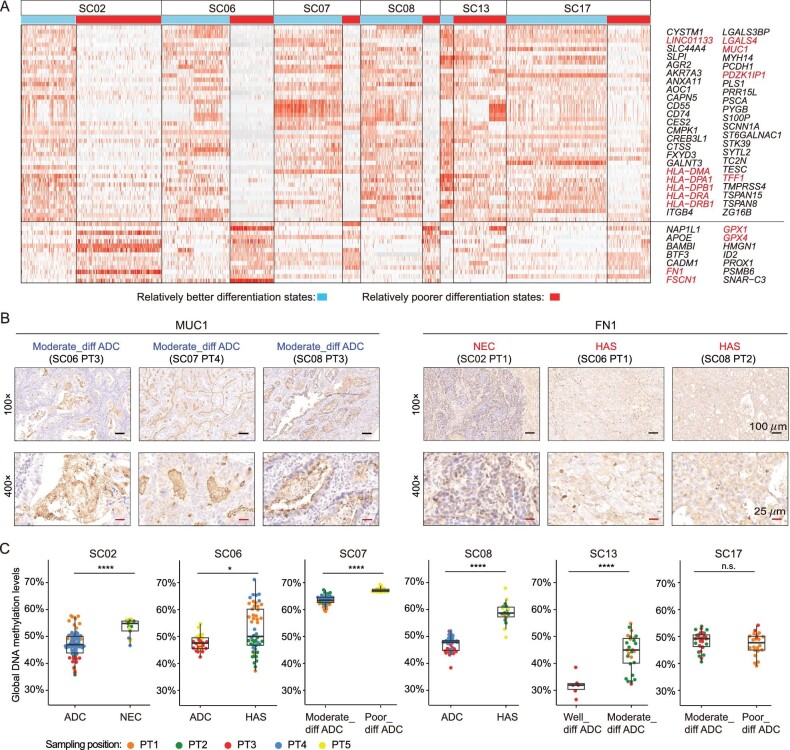
Molecular features of different differentiation states of cancer cells. (A) The heatmap shows the relative expression levels of DEGs between two major differentiation states within each mGC patient. The DEGs which were shared by more than 5/6 mGC patients were shown. (B) The IHC staining of MUC1 and FN1 protein of some representative positions of mGC patients. (C) The global DNA methylation levels (WCG, W denotes A or T) of cancer cells with different differentiation states, of six mGC patients. Wilcoxon rank-sum test, ****P-value < 0.0001, ***P-value < 0.001, **P-value < 0.01, *P-value < 0.05. n.s., not significant.

As the transcriptomic clusters within each mGC patient can be divided into two major differentiation states (Fig. 3B), we searched DEGs between the two major differentiation states (Table S5). To eliminate inter-patient heterogeneities, we performed the DEG analyses within the same mGC patient. We found that the DEGs were heterogeneous among patients in general, but can converge on some important pathways (Fig. S8A). For example, pathways involved in cell cycle and stress response were significantly enriched using the up-regulated genes of cancer cells with poorer differentiation states within each mGC patient (Fig. S8A). We also identified some shared DEGs among the majority of mGC patients (≥5/6) (Fig. 4A). For example, mucin 1 (*MUC1*), which is a member of the mucin family, significantly down-regulated in ADC with poorer differentiation states than in ADC with better differentiation states, and significantly down-regulated in non-ADC (NEC and HAS) compared with ADC within the same mGC patient (Fig. 4A), which was also validated by IHC staining at protein levels (Fig. 4B). Additionally, some of the up-regulated genes of cancer cells with poorer differentiation states were significantly associated with poorer GC prognosis using TCGA data (Fig. S8B). For example, the fibronectin 1 gene (*FN1*) was significantly up-regulated in non-ADC (NEC and HAS) compared to ADC (Fig. 4A), and was validated by IHC staining at protein level (Fig. 4B).

We further explored whether epigenetic regulation participated in regulating the DEGs between different differentiation states. At the global level, we found that cancer cells with poorer differentiation states significantly up-regulated global DNA methylation levels compared to those with better differentiation states (but still lower than normal_epi_stomach) in PTs of 5/6 mGC patients (Fig. 4C). Then we searched for the DEGs with altered DNA methylation and/or chromatin accessibility levels in their promoter regions within each mGC patient (Table S6). We found that the gene lists were also highly heterogeneous among different mGC patients, but could converge on some shared pathway terms (Fig. S9A). For example, pathways involving digestion, immune response and cell adhesion were enriched using the down-regulated genes of cancer cells with poorer differentiation states, which were potentially regulated by up-regulated promoter DNA methylation levels, suggesting more malignant characteristics of cancer cells with poorer differentiation states. Additionally, a small number of marker genes shared by multiple mGC patients (≥3/6) were identified. For example, the promoter DNA methylation levels of *MUC1* increased significantly in non-ADC (NEC and HAS) compared to ADC, or in ADC with poorer differentiation states compared to ADC with better differentiation states, for the 4/6 mGC patients, and the promoter chromatin accessibility levels of *MUC1* also showed a decreased tendency in the four patients (although not statistically significant in two patients), indicating that the decreased expression levels of *MUC1* were likely to be regulated by both decreased chromatin accessibility and increased DNA methylation levels (Fig. S9B). Some other genes, such as *TFF1, LGALS4, LINC01133* and *PDZK1IP1* showed a similar tendency in multiple mGC patients (Table S6). Additionally, the increased expression levels of *CMTM3* may be regulated by increased chromatin accessibility and decreased DNA methylation levels in the promoter region for 5/6 mGC patients, and were also associated with poor prognosis (Fig. S9B and C). The increased expression levels of *FN1* may be regulated by increased promoter chromatin accessibility levels for 5/6 mGC patients (not statistically significant in three patients) (Fig. S9B). Hence, here we found that the differentiation states of cancer cells may be regulated by epigenetic changes to some extent.

### Gastric cancers with different differentiation states displayed different immune states

We noticed that some MHC class II genes (*HLA-DMA, HLA-DPA1, HLA-DPB1, HLA-DRA, HLA-DRB1*) were significantly down-regulated in cancer cells with relatively poor differentiation states compared with those with better differentiation states for 5/6 mGC patients (Fig. 4A and Fig. S10A; Table S5). The down-regulated genes of cancer cells with poorer differentiation states within each mGC patient were also significantly enriched in immune-related pathways (Fig. S8A). The results indicated less immune activity and probably lower antigen-presenting activities of the non-ADC (NEC and HAS) cancer cells.

**Figure 5.**
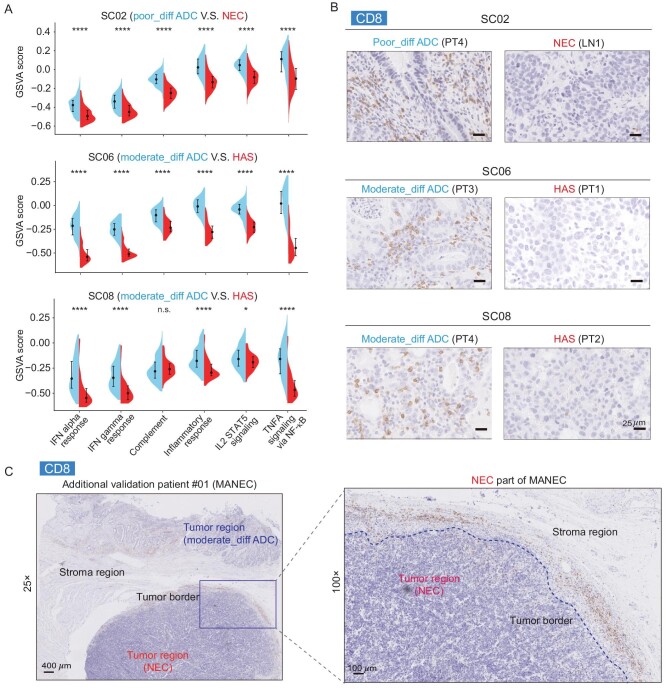
Cancer cells with different differentiation states show different immune states. (A) The GSVA scores of immune-related pathways in the hallmark pathways. The median values and interquartile ranges are shown. Wilcoxon rank-sum test, ****P-value < 0.0001, ***P-value < 0.001, **P-value < 0.01, *P-value < 0.05. n.s., not significant. (B) IHC staining of CD8 protein for some representative sampling positions of mGCs. Scale bars, 25 μm. (C) IHC staining of CD8 protein for an additional validation patient with MANEC.

To further assess the differences in immune states between different differentiation states of cancer cells, we calculated the gene set variation analysis (GSVA) scores [19] of immune-related pathways in hallmark gene sets for each mGC patient. We found that the immune scores of non-ADC (NEC and HAS) significantly decreased compared with those in ADC within the same mGC patient (Fig. 5A). Moreover, we performed IHC staining of mGCs to explore the immune infiltration states of cancer samples with different differentiation states. We found that CD8^+^ T cells rarely infiltrated into the non-ADC (NEC and HAS) tumor part of mGCs (Fig. 5B, Fig. S10B), and only surrounded the tumor border (Fig. 5C), which was in contrast with the ADC counterparts. The data suggested that the non-ADC (NEC and HAS) GCs were immune-excluded and escaped immune surveillance, which could partially explain the poor prognosis of non-ADC (NEC and HAS).

## DISCUSSION

In this study, our single-cell multiomics sequencing and multi-regional sampling strategy enabled the identification of prevalent molecular alterations of cancer cells, and the dissection of the ITH and multiomics molecular features of different differentiation states of cancer cells, providing novel insights into the molecular basis of GC.

First, we identified common transcriptomic alterations of GC cells compared with normal gastric epithelial cells, and revealed that GC cells aberrantly and prevalently up-regulated many highly expressed genes of normal_epi_colon, and partially down-regulated many highly expressed genes of normal_epi_stomach. Moreover, we found that promoter DNA methylation and chromatin accessibility levels may play an important role in regulating the transcriptomic alterations of cancer cells.

Second, we delineated the DNA methylation alteration map of GC cells at single-cell resolution. DNA hypomethylation can influence genome integrity by de-repressing repeat elements [18]. We detected strong DNA demethylation of repeat elements in cancer cells, and strong expression of L1 ORF1p protein in GC cells, suggesting that the retrotransposon L1 may be aberrantly reactivated in GC and destruct genome integrity. Additionally, we identified prevalent hypermethylated or hypomethylated promoters of marker genes in cancer cells, providing candidate DNA methylation biomarkers for human GC.

Third, we systematically revealed the complex relationship between the genome (SCNAs), epigenome and transcriptome of mGC using parallel single-cell multiomic sequencing data. The global DNA methylation heterogeneities mainly existed among different genetic lineages of GC cells in the same patient, whereas the global DNA methylation characteristics were in general maintained within the same genetic lineage during tumorigenesis and progression. Additionally, although copy number alterations can influence gene expression by altering gene dosage, disrupting gene structures, or affecting regulatory regions [20], the relationships between genetic lineages and transcriptomic clusters of cancer cells were complex. One genetic lineage of cancer cells can correspond to multiple transcriptomic clusters, and different genetic lineages can correspond to the same transcriptomic cluster.

Fourth, with the advantages of multi-regional sampling and single-cell multiomics sequencing, we could identify the different differentiation states for cancer cells within each mGC patient, and further revealed the DEGs, signaling pathways and potential epigenetic regulations of different differentiation states. Previous studies have found that global DNA hypomethylation in cancer cells can activate immune responses [21]. Our data showed that non-ADC cancer cells had higher global DNA methylation levels than ADC cancer cells within the same patient (although their DNA methylation levels were still lower than normal_epi_stomach), as well as down-regulated immune responses and poorer infiltration of CD8^+^ T cells, which indicated that these tumors escape immune surveillance and are probably resistant to immunotherapy. DNA demethylation drugs, such as 5-Aza-CdR, may be good candidates for helping immune systems to attack cancer cells [22] in GC patients, especially for non-ADC (NEC and HAS), which needs further investigation.

We acknowledge that our study has limitations in terms of the number of single cells analyzed due to the relatively high cost and low throughput of the scTrio-seq3 technique. However, we performed multi-regional sampling of each tumor (including 85 sampling positions) and found extensive spatial heterogeneity of multiomic features, suggesting that the multi-regional sampling strategy is important for capturing ITH. Although the throughput of several commercialized methods is higher (such as 10x Genomics), these methods have not analyzed the SCNAs, DNA methylome, chromatin accessibility and transcriptome simultaneously in a single cell to date, and therefore cannot meet the needs of our study. Further investigations to develop multiomics sequencing with reduced cost and higher throughput will be helpful.

In summary, our study revealed the molecular characteristics of intratumoral heterogeneities and different differentiation states of GC at multiomic levels, providing a deeper understanding of the molecular basis of human GC.

## MATERIALS AND METHODS

Detailed materials and methods are available in the Supplementary data.

## DATA AVAILABILITY

All the sequencing data have been submitted to the genome sequence archive (GSA) database under the accession number HRA001689 (https://ngdc.cncb.ac.cn/gsa/) under controlled access due to human data privacy. Requests should be sent to the lead contact F.T. (tangfuchou@pku.edu.cn).

## Supplementary Material

nwad094_Supplemental_Files
