# Supplementary material for: Integrative single-cell multiomics analyses dissect molecular signatures of intratumoral heterogeneities and differentiation states of human gastric cancer
Source: Natl Sci Rev. 2023 Apr 11;10(6):nwad094. doi: 10.1093/nsr/nwad094 (PMC10281500; doi:10.1093/nsr/nwad094)
Supplement: nwad094_Supplemental_Files [file nwad094_supplemental_files.zip › Supplementary methods V20230409.pdf]

METHODS

Clinical sample collection

This study was approved by the Ethics Committee of Peking University Third Hospital (License No. IRB00006761-M2016170). The informed consents were signed by all of the involved patients preoperatively. Multi-regional sampling was performed for 14 patients with gastric cancer. Normal adjacent tissues, marginal and central areas of primary tumors, and lymph node metastases were sampled.

Single cell preparation

Fresh tissue samples were cut into small pieces, and then digested into single-cell suspension using 1.5 mg/mL collagenase type II (Gibco, 17101015) and 1.5 mg/mL collagenase type IV (Gibco, 17104019). After filtered by 40 μm filters (BD, 352340), the cells were centrifuged and resuspended in human red blood cell lysis (TIANDZ, 90309-250) for 1 min at room temperature. Because we mainly focus on cancer cells, we enriched cancer cells using EPCAM antibody (BioLegend, 324208; Miltenyi, 130-061-101) or tumor cell isolation kit (Miltenyi, 130-108-339), or depleted the leukocytes using CD45 antibody (Miltenyi, 130-045-801; BioLegend, 368522) via MACS or FACS. Although cancer cells may undergo epithelial-to-messenthymal transition (EMT), recent study found cancer cells are in p-EMT (partial EMT) state remaining the expression of EPCAM (*1*). The sorted single cells were randomly picked into PCR tubes using the mouth pipette for all the patients.

Single-cell multiomic sequencing (scTrio-seq3) experiment

The sorted single cells were picked into the lysis buffer which contained GpC methyltransferase (NEB, M0227L). After being gently vortexed for 20 seconds, the cells were performed *in vitro* methylation at 37°C for 20 min and the GpC methyltransferase was heat inactivated at 65 ℃ for 20 min. We used the strategy of scTrio-seq2 (*2*) to separate the nucleus and mRNA in cytoplasm. The supernatants contained mRNA were transferred into Smart-seq2 reverse transcription (RT) mixture (*3*) with the depletion of first-strand buffer and with the addition of 4.3 μL primer mix (1mM dNTPs and 3 μM barcode RT primers), and then were incubated at 25°C for 5 min, 42°C for 60 min, 50°C for 30 min, and 70°C for 10 min for the

synthesis of cDNA. Then, we add 15 μL Smart-seq2 PCR mix to amplify cDNA. The cDNA of cells with different barcodes were pooled together and the construction of the library were performed as previously study (*4*). The single-cell genomic DNA library construction for analyzing DNA methylome and chromatin accessibility was performed according to our previous published paper (*5*, *6*). Briefly, the bead-coated nuclei were resuspended by protein digestion buffer containing 0.5 μL of protease K (Zymo, D5044) and 2.5 μL of M-digestion buffer (Zymo, D5044) and were then incubated at 50°C for 1 hour to release the genomic DNA. Bisulfite conversion was performed on the gDNA using the EZ-96 DNA Methylation-Direct™ Mag Prep Kit (Zymo, D5044). The random priming and extension was 4 rounds in total. After random priming, we performed 0.8× Agencourt Ampure XP beads (Beckman Coulter, A63882) directly. Libraries were amplified by PCR of 16 cycles. Finally, the libraries were purified with 0.8× Agencourt Ampure XP beads twice and the purified libraries were sequenced on the Illumina HiSeq 4000 platform.

Hematoxylin-eosin (H&E) staining and immunohistochemistry (IHC) staining

Fresh tissues were fixed in neutral buffered formalin overnight and embedded in paraffin. Subsequently, H&E staining and immunohistochemistry staining were performed on 5-μm thick slides according to standard procedures. For IHC staining, the sections were deparaffinized and hydrated sequentially, and the endogenous peroxidase activity was inactivated by 3% H_2O_2. Then, the sections were processed by pH 6.0 citrate buffer for antigen retrieval and blocked by BSA. The sections were incubated with the primary antibody at 37°C for 2 h. After washed by PBS for three times, the HRP-conjugated secondary antibody were added and incubated at 37°C for 30 min. DAB was used to reveal the color and hemsatoxylin was used to counterstain. Finally, the images were captured by NanoZoomer SQ, and assessed by two independent pathologists. The antibodies used in IHC are listed as following: anti-MUC1 antibody (Abcam, ab70475), anti-FN1 antibody (Abcam, ab2413), anti-LINE-1 ORF1p antibody (Abcam, ab245249), and anti-CD8 antibody (ZSGB-BIO, ZA-0508).

Processing of the single-cell RNA-seq data from the multiomics sequencing

UMI-tools (version 0.5.5) (*7*) were used to extract single-cell information from the raw sequencing data, including the 8-nt cell barcodes followed by 8-nt UMIs (random nucleotides) at the beginning of Read 2. Then, fastp (version 0.19.8) (*8*) with default parameters and custom scripts were used to trim low-quality bases, adaptors, poly A and TSO sequences (AAGCAGTGGTATCAACGCAGAGTAC) in Read 1. The trimmed reads were mapped to the human reference genome hg19 using STAR (version 2.6.1d) (*9*). Afterward, featureCounts tool of subread (version 1.6.3) (*10*) were used to align the mapped reads to RefSeq genes. Then, we used UMI-tools (version 0.5.5) to count UMI numbers of each gene. We filtered out cells with less than 1,500 RefSeq genes detected. With high-quality single-cell transcriptome data (on average 5,966 genes detected in one cell), we performed the uniform manifold approximation and projection (UMAP) analysis of normal and cancer epithelial cells using Seurat (version 3.1.1) (*11*). A total of 3,000 variable genes were selected using the "FindVariableFeatures" function. To explore the shared molecular alterations during tumorigenesis, we compared the significant DEGs between cancer cells and normal_epi_stomach using the "FindMarkers" function in Seurat (version 3.1.1) under very stringent cutoff (fold change >2, percentage of expressed cells >80%, adjusted P-value <0.05, Wilcoxon rank-sum test). We compared the scRNA-seq data of normal_epi_stomach and normal_epi_colon using stringent cutoff (fold change >2, percentage of expressed cells >70%, adjusted P-value <0.05, Wilcoxon rank-sum test). The pathway enrichment was performed using Metascape database (*12*). The gene expression matrix ($\log_2$(TPM/10 +1)) of normal_epi_stomach and cancer cells were processed using the "normalize.quantiles" function in the R package "preprocessCore" and then were used in the PCA analyses using "prcomp" function in R. Because PC2 was dominated by individual differences between SC17 and others, it was not considered in subsequent analyses.

Processing of single-cell DNA-seq data from the multiomics sequencing

Firstly, the Trim Galore tool (version 0.4.4) (http://www.bioinformatics.babraham.ac.uk/projects/trim_galore/) was used to trim low-quality bases and random primers (6-nt). Afterwards, the trimmed reads was mapped to human reference genome hg19 as well as lambda DNA reference genome using Bismark

(version 0.7.6) (*13*). Then, we removed the duplication reads using Samtools (version 0.1.18) (*14*). We used strict quality control standard, and only cells with mapping ratio ≥5%, whole-genome coverage ratio ≥4%, number of WCG sites ≥800,000, number of GCH sites ≥5,000,000, and CT conversion rate ≥98% were used in further analyses (Table S1).

We used the methylation levels in WCG sites to represent the endogenous DNA methylation levels, and the *in vitro* DNA methylation levels of GCH site to represent chromatin accessibility levels. For each WCG or GCH site, only those with methylation levels >0.9 or <0.1 were used in further analyses. We defined the promoter regions as the upstream 1-kb regions and downstream 0.5-kb regions of gene transcription start sites (TSSs). The repeat element regions were downloaded from UCSC genome browser (http://genome.ucsc.edu/). Only the tiles covered by ≥3 WCG or ≥3 GCH sites were used when calculating the DNA methylation or chromatin accessibility levels of genomic elements and 1-kb consecutive tiles. When calculating the whole-genome DNA methylation or chromatin accessibility level of each individual cell, we firstly calculated the mean levels of each 1-kb tile, and then used the average levels of the tiles to represent global levels. The function 'cmdscale' in R was used to perform classical multidimensional scaling (MDS) of promoter DNA methylation levels and chromatin accessibility levels. Only the promoters covered by ≥3 WCG or ≥3 GCH sites were used in each individual cell, and only the promoters covered by ≥50% cells (for WCG) or 20% cells (for GCH) were used in the MDS analysis. In addition, to search the general hypermethylated promoters and hypomethylated promoters of all gastric cancer patients, we compared the promoter DNA methylation levels between cancer cells of each patient and all normal epithelial cells using very strict criteria (Wilcoxon rank-sum test, P-value < 0.05, fold change > 2, absolute difference of DNA methylation levels between two groups > 0.5, SD within each group < 0.25).

We performed SCNA estimation of all sequenced cells using the DNA part of single-cell multiomics sequencing. We deduced the SCNA profiles in individual cells using the same process as reported in the colorectal cancer (*2, 15*). Normal gastric epithelial cells were used as control for normalization.

Distinguish cancer cells from normal epithelial cells using single-cell multiomics sequencing data

After stringent filtering and quality control for the single-cell multiomics sequencing data, the data of 4,657 single cells passed initial quality control, including RNA data of 4,206 cells and DNA data of 2,036 cells. Cancer cells within the same patient had cancer-specific SCNA pattern and normal cells have nearly euploid genome. RNA UMAP analysis can also distinguish normal cells and cancer cells. Combining SCNA and UMAP information, we can accurately and strictly classify cells as 4 major cell types: normal epithelial cells sampled from NAT (euploid without SCNAs), cancer cells sampled from tumor tissues (with cancer-specific SCNAs), stromal cells sampled from tumor tissues (immune cells, fibroblasts, endothelial cells, etc.) and normal epithelial cells mixed in tumor tissues. Then, the first two types (normal epithelial cells sampled from NAT and cancer cells sampled from tumor tissues) were mainly used in the following analyses (Table S1).

Overall survival analysis

We performed the overall survival analyses of TCGA STAD (stomach adenocarcinoma) cohort using website server (http://gepia2.cancer-pku.cn/) (*16*). We used quantiles as cutoff for high expression group and low expression group.

REFERENCES

1. S. V. Puram, I. Tirosh, A. S. Parikh, A. P. Patel, K. Yizhak, S. Gillespie, C. Rodman, C. L. Luo, E. A. Mroz, K. S. Emerick, D. G. Deschler, M. A. Varvares, R. Mylvaganam, O. Rozenblatt-Rosen, J. W. Rocco, W. C. Faquin, D. T. Lin, A. Regev, B. E. Bernstein, Single-cell transcriptomic analysis of primary and metastatic tumor ecosystems in head and neck cancer. *Cell*. **171**, 1611-1624.e24 (2017).

2. S. Bian, Y. Hou, X. Zhou, X. Li, J. Yong, Y. Wang, W. Wang, J. Yan, B. Hu, H. Guo, J. Wang, S. Gao, Y. Mao, J. Dong, P. Zhu, D. Xiu, L. Yan, L. Wen, J. Qiao, F. Tang, W. Fu, Single-cell multiomics sequencing and analyses of human colorectal cancer. *Science*. **362**, 1060–1063 (2018).

3. S. Picelli, O. R. Faridani, Å. K. Björklund, G. Winberg, S. Sagasser, R. Sandberg, Full-length RNA-seq from single cells using Smart-seq2. *Nat. Protocols*. **9**, 171–181 (2014).

4. J. Dong, Y. Hu, X. Fan, X. Wu, Y. Mao, B. Hu, H. Guo, L. Wen, F. Tang, Single-cell RNA-seq analysis unveils a prevalent epithelial/mesenchymal hybrid state during mouse organogenesis. *Genome Biol*. **19**, 31 (2018).

5. X. Fan, P. Lu, H. Wang, S. Bian, X. Wu, Y. Zhang, Y. Liu, D. Fu, L. Wen, J. Hao, F. Tang, Integrated single-cell multiomics analysis reveals novel candidate markers for prognosis in human pancreatic ductal adenocarcinoma. *Cell Discov*. **8**, 13 (2022).

6. Y. Wang, H. Xie, X. Chang, W. Hu, M. Li, Y. Li, H. Liu, H. Cheng, S. Wang, L. Zhou, D. Shen, S. Dou, R. Ma, Y. Mao, H. Zhu, X. Zhang, Y. Zheng, X. Ye, L. Wen, K. Kee, H. Cui, F. Tang, Single-cell dissection of the multiomic landscape of high-grade serous ovarian cancer. *Cancer Research*. **82**, 3903–3916 (2022).

7. T. Smith, A. Heger, I. Sudbery, UMI-tools: modeling sequencing errors in Unique Molecular Identifiers to improve quantification accuracy. *Genome Res*. **27**, 491–499 (2017).

8. S. Chen, Y. Zhou, Y. Chen, J. Gu, fastp: an ultra-fast all-in-one FASTQ preprocessor. *Bioinformatics*. **34**, i884–i890 (2018).

9. A. Dobin, C. A. Davis, F. Schlesinger, J. Drenkow, C. Zaleski, S. Jha, P. Batut, M. Chaisson, T. R. Gingeras, STAR: ultrafast universal RNA-seq aligner. *Bioinformatics*. **29**, 15–21 (2013).

10. Y. Liao, G. K. Smyth, W. Shi, featureCounts: an efficient general purpose program for assigning sequence reads to genomic features. *Bioinformatics*. **30**, 923–930 (2014).

11. T. Stuart, A. Butler, P. Hoffman, C. Hafemeister, E. Papalexi, W. M. Mauck, Y. Hao, M. Stoeckius, P. Smibert, R. Satija, Comprehensive integration of single-cell data. *Cell*. **177**, 1888-1902.e21 (2019).

12. Y. Zhou, B. Zhou, L. Pache, M. Chang, A. H. Khodabakhshi, O. Tanaseichuk, C. Benner, S. K. Chanda, Metascape provides a biologist-oriented resource for the analysis of systems-level datasets. *Nat Commun*. **10**, 1523 (2019).

13. F. Krueger, S. R. Andrews, Bismark: a flexible aligner and methylation caller for Bisulfite-Seq applications. *Bioinformatics*. **27**, 1571–1572 (2011).

14. H. Li, B. Handsaker, A. Wysoker, T. Fennell, J. Ruan, N. Homer, G. Marth, G. Abecasis, R. Durbin, The Sequence Alignment/Map format and SAMtools. *Bioinformatics*. **25**, 2078–2079 (2009).

15. Y. Zhou, S. Bian, X. Zhou, Y. Cui, W. Wang, L. Wen, L. Guo, W. Fu, F. Tang, Single-cell multiomics sequencing reveals prevalent genomic alterations in tumor stromal cells of human colorectal cancer. *Cancer Cell*. **38**, 818-828.e5 (2020).

16. Z. Tang, B. Kang, C. Li, T. Chen, Z. Zhang, GEPIA2: an enhanced web server for large-scale expression profiling and interactive analysis. *Nucleic Acids Res*. **47**, W556–W560 (2019).

Table S1. Summary of the single-cell multiomics data

Table S2. The DEGs between cancer cells and normal gastric epithelial cells

Table S3. The differentially methylated promoters between cancer cells and normal gastric epithelial cells

Table S4. The DEGs of transcriptome clusters for each mGC patient

Table S5. The DEGs between the two major differentiation states within each mGC patient

Table S6. The DEGs associated with altered promoter DNA methylation or chromatin accessibility levels
