# Supplementary material for: Integrative single-cell multiomics analyses dissect molecular signatures of intratumoral heterogeneities and differentiation states of human gastric cancer
Source: Natl Sci Rev. 2023 Apr 11;10(6):nwad094. doi: 10.1093/nsr/nwad094 (PMC10281500; doi:10.1093/nsr/nwad094)
Supplement: nwad094_Supplemental_Files [file nwad094_supplemental_files.zip › Supplementary figures and legend V20230409.pdf]

Figure S1

A

No.	Patient	Gender	Age at Diagnosis	Anatomic Region	AJCC Stage	Lauren Classification	MSI Status	Sampling Regions NAT	PT	LN
1	SC02	Male	62	Cardia	IIIC	Mixed	-	●	▲▲▲▲▲	■■■■■■
2	SC06	Male	71	Cardia	IIB	Mixed	-	●	▲▲▲▲▲	■■■
3	SC07	Female	71	Body	IIB	Mixed	+	●	▲▲▲▲	■
4	SC08	Male	61	Cardia	IIIB	Mixed	-	●	▲▲▲▲▲	■■
5	SC13	Female	66	Antrum	IIIB	Mixed	-	●	▲▲▲	■
6	SC18	Male	71	Body	IIIC	Mixed	-	●	▲▲	■■
7	SC03	Male	63	Body	IIIC	Diffuse	-	●	▲▲▲▲▲	■■■■
8	SC05	Male	74	Antrum	IIIA	Diffuse	-	●	▲▲▲▲▲	■■
9	SC14	Male	62	Body	IIIC	Diffuse	-	●	▲▲▲	*
10	SC15	Male	85	Antrum	IIB	Diffuse	-	●	▲▲	*
11	SC10	Male	64	Antrum	IIB	Intestinal	-	●	▲▲▲	□□
12	SC11	Male	85	Antrum	IIA	Intestinal	-	●	▲	*
13	SC16	Male	67	Antrum	IIIC	Intestinal	-	●	▲▲	■
14	SC17	Female	68	Body	IIB	Intestinal	-	●	▲▲▲	■

Annotation: ●▲■ Sampled □ Sampled but lacking cancer cells *Absent

B

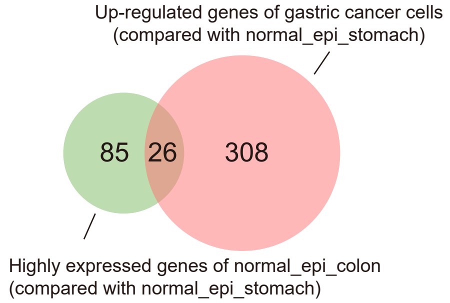

C

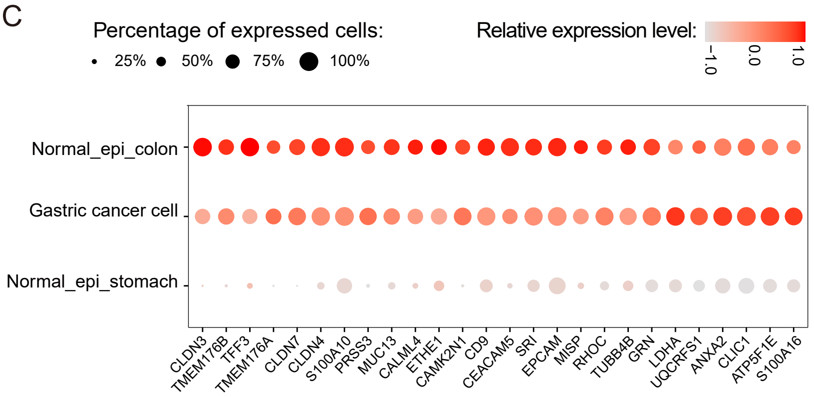

D

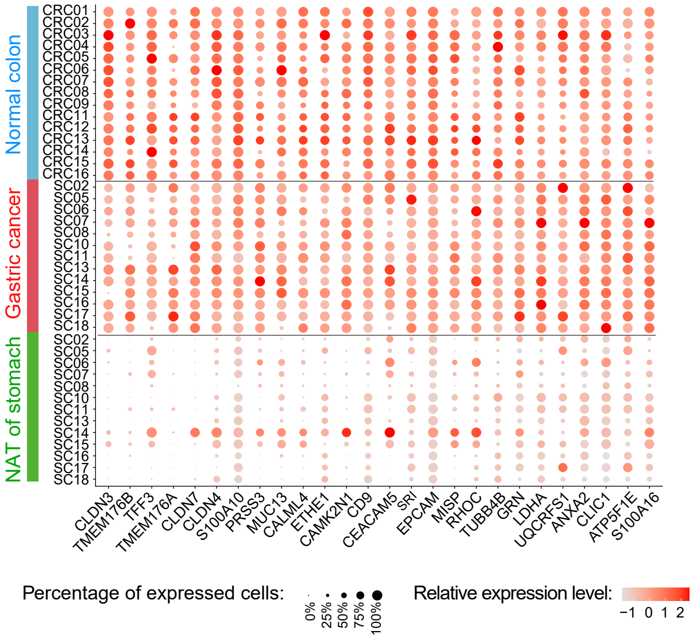

E

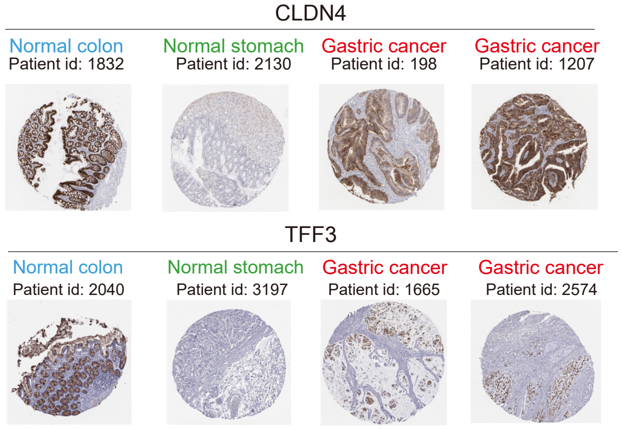

F

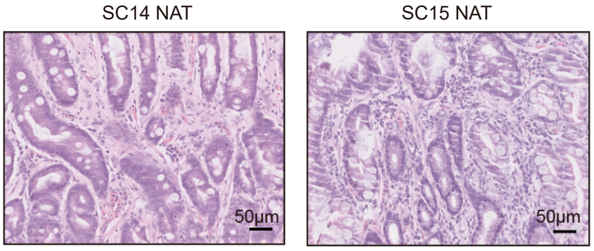

G

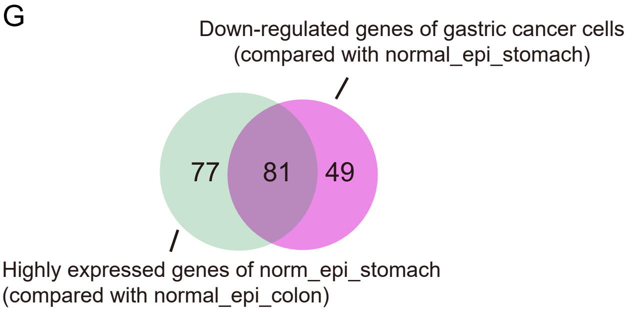

H

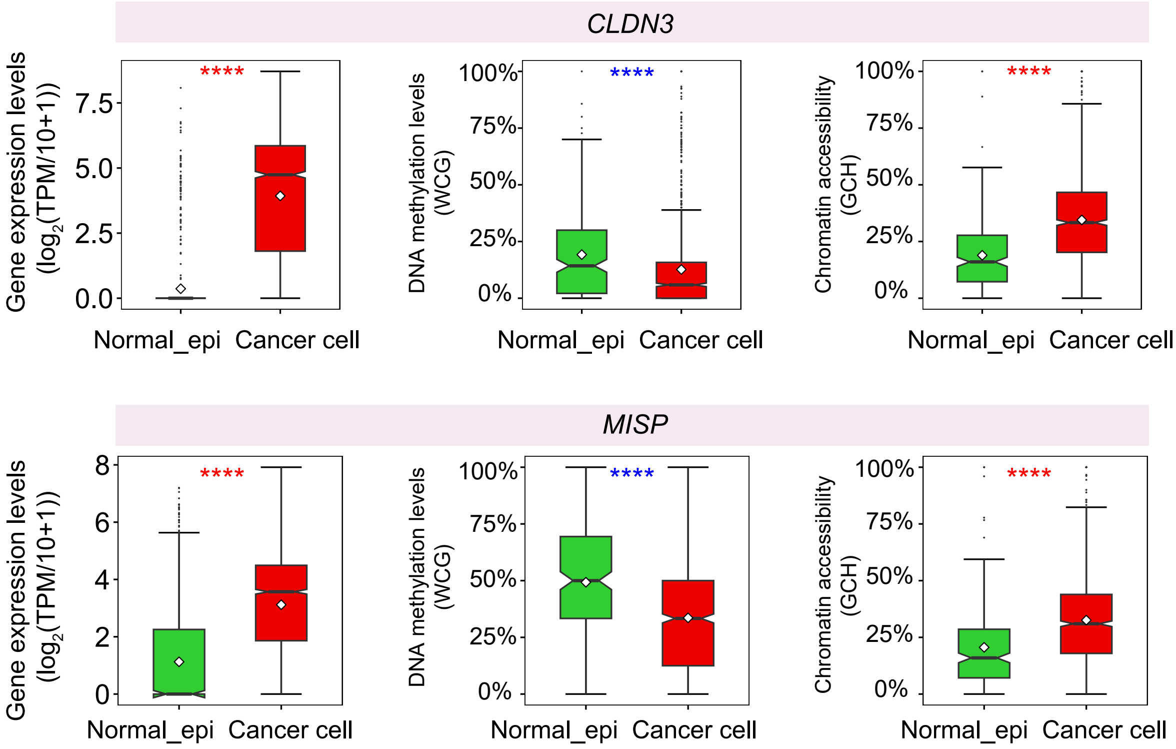
CLDN3
Gene expression levels (log2(TPM/10+1))
7.5
5.0
2.5
0.0
****
Normal_epi
Cancer cell
DNA methylation levels (WCG)
100%
75%
50%
25%
0%
****
Normal_epi
Cancer cell
Chromatin accessibility (GCH)
100%
75%
50%
25%
0%
****
Normal_epi
Cancer cell
MISP
Gene expression levels (log2(TPM/10+1))
8
6
4
2
0
****
Normal_epi
Cancer cell
DNA methylation levels (WCG)
100%
75%
50%
25%
0%
****
Normal_epi
Cancer cell
Chromatin accessibility (GCH)
100%
75%
50%
25%
0%
****
Normal_epi
Cancer cell

Figure S2

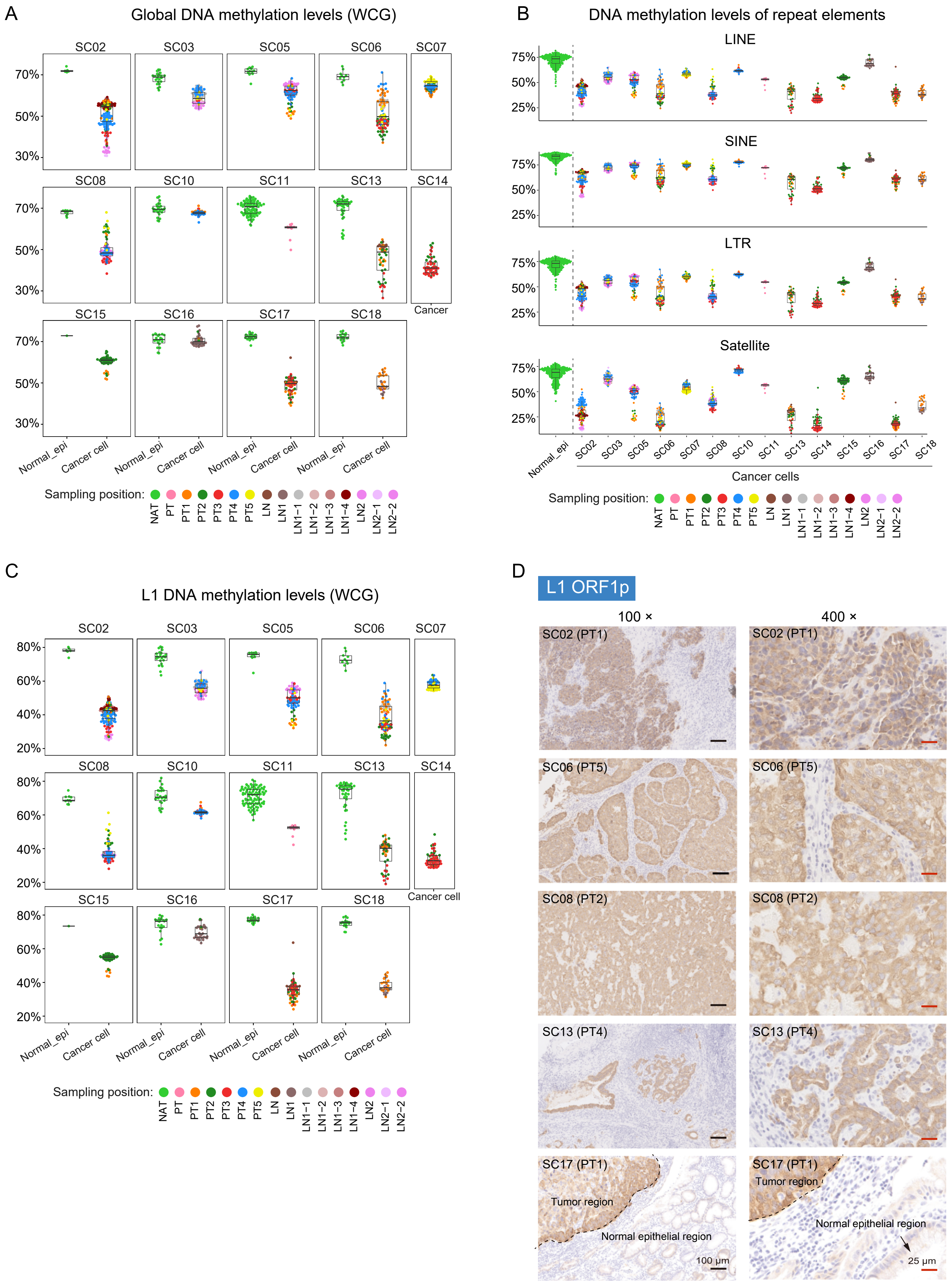

A
Global DNA methylation levels (WCG)
SC02 SC03 SC05 SC06 SC07
SC08 SC10 SC11 SC13 SC14
SC15 SC16 SC17 SC18
70% 50% 30%
Cancer
Normal_epi Cancer cell
Sampling position: NAT PT PT1 PT2 PT3 PT4 PT5 LN LN1 LN1−1 LN1−2 LN1−3 LN1−4 LN2 LN2−1 LN2−2
B
DNA methylation levels of repeat elements
LINE
SINE
LTR
Satellite
75% 50% 25%
Normal_epi SC02 SC03 SC05 SC06 SC07 SC08 SC10 SC11 SC13 SC14 SC15 SC16 SC17 SC18
Cancer cells
Sampling position: NAT PT PT1 PT2 PT3 PT4 PT5 LN LN1 LN1−1 LN1−2 LN1−3 LN1−4 LN2 LN2−1 LN2−2
C
L1 DNA methylation levels (WCG)
SC02 SC03 SC05 SC06 SC07
SC08 SC10 SC11 SC13 SC14
SC15 SC16 SC17 SC18
80% 60% 40% 20%
Cancer cell
Normal_epi Cancer cell
Sampling position: NAT PT PT1 PT2 PT3 PT4 PT5 LN LN1 LN1−1 LN1−2 LN1−3 LN1−4 LN2 LN2−1 LN2−2
D
L1 ORF1p
100 ×
400 ×
SC02 (PT1)
SC06 (PT5)
SC08 (PT2)
SC13 (PT4)
SC17 (PT1)
Tumor region
Normal epithelial region
100 μm
25 μm

Figure S3

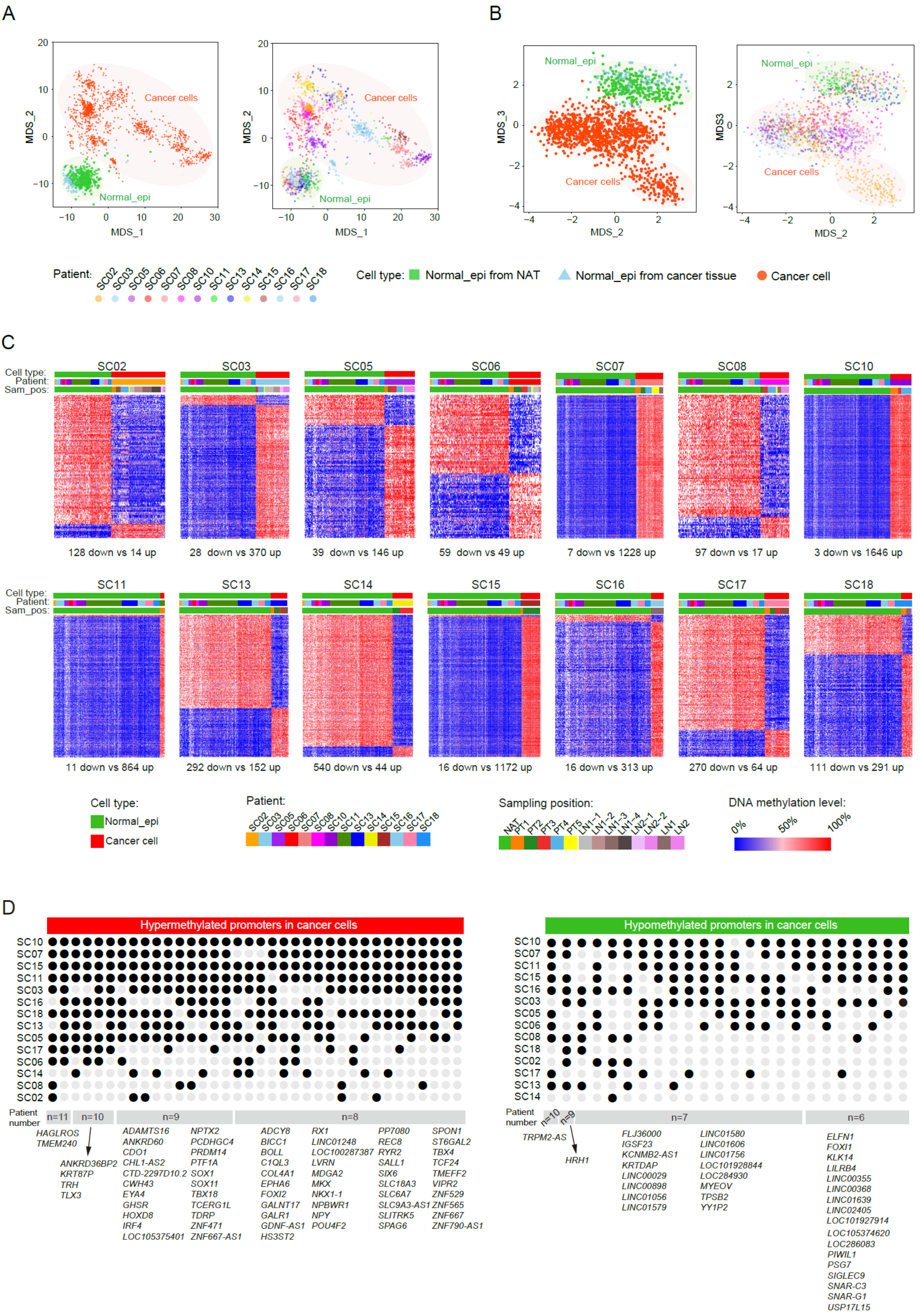
A
MDS_2
MDS_1
Cancer cells
Normal_epi
B
MDS_3
MDS_2
MDS3
Normal_epi
Cancer cells
Patient:
SC02 SC03 SC05 SC06 SC07 SC08 SC10 SC11 SC13 SC14 SC15 SC16 SC17 SC18
Cell type: Normal_epi from NAT
Normal_epi from cancer tissue
Cancer cell
C
Cell type:
Patient:
Sam_pos:
SC02
128 down vs 14 up
SC03
28 down vs 370 up
SC05
39 down vs 146 up
SC06
59 down vs 49 up
SC07
7 down vs 1228 up
SC08
97 down vs 17 up
SC10
3 down vs 1646 up
SC11
11 down vs 864 up
SC13
292 down vs 152 up
SC14
540 down vs 44 up
SC15
16 down vs 1172 up
SC16
16 down vs 313 up
SC17
270 down vs 64 up
SC18
111 down vs 291 up
Cell type:
Normal_epi
Cancer cell
Patient:
SC02 SC03 SC05 SC06 SC07 SC08 SC10 SC11 SC13 SC14 SC15 SC16 SC17 SC18
Sampling position:
NAT PT1 PT2 PT3 PT4 PT5 LN1-1 LN1-2 LN1-3 LN1-4 LN2-1 LN2-2 LN1 LN2
DNA methylation level:
0% 50% 100%
D
Hypermethylated promoters in cancer cells
SC10 SC07 SC15 SC11 SC03 SC16 SC18 SC13 SC05 SC17 SC06 SC14 SC08 SC02
Patient number
n=11 n=10 n=9 n=8
HAGLROS
TMEM240
ANKRD36BP2
KRT87P
TRH
TLX3
ADAMTS16
ANKRD60
CDO1
CHL1-AS2
CTD-2297D10.2
CWH43
EYA4
GHSR
HOXD8
IRF4
LOC105375401
NPTX2
PCDHGC4
PRDM14
PTF1A
SOX1
SOX11
TBX18
TCERG1L
TDRP
ZNF471
ZNF667-AS1
ADCY8
BICC1
BOLL
C1QL3
COL4A1
EPHA6
FOXI2
GALNT17
GALR1
GDNF-AS1
HS3ST2
RX1
LINC01248
LOC100287387
LVRN
MDGA2
MKX
NKX1-1
NPBWR1
NPY
POU4F2
PP7080
REC8
RYR2
SALL1
SIX6
SLC18A3
SLC6A7
SLC9A3-AS1
SLITRK5
SPAG6
SPON1
ST6GAL2
TBX4
TCF24
TMEFF2
VIPR2
ZNF529
ZNF565
ZNF667
ZNF790-AS1
Hypomethylated promoters in cancer cells
SC10 SC07 SC11 SC15 SC16 SC03 SC05 SC06 SC08 SC18 SC02 SC17 SC13 SC14
Patient number
n=10 n=9 n=7 n=6
TRPM2-AS
HRH1
FLJ36000
IGSF23
KCNMB2-AS1
KRTDAP
LINC00029
LINC00898
LINC01056
LINC01579
LINC01580
LINC01606
LINC01756
LOC101928844
LOC284930
MYEOV
TPSB2
YY1P2
ELFN1
FOXI1
KLK14
LILRB4
LINC00355
LINC00368
LINC01639
LINC02405
LOC101927914
LOC105374620
LOC286083
PIWIL1
PSG7
SIGLEC9
SNAR-C3
SNAR-G1
USP17L15

Figure S4

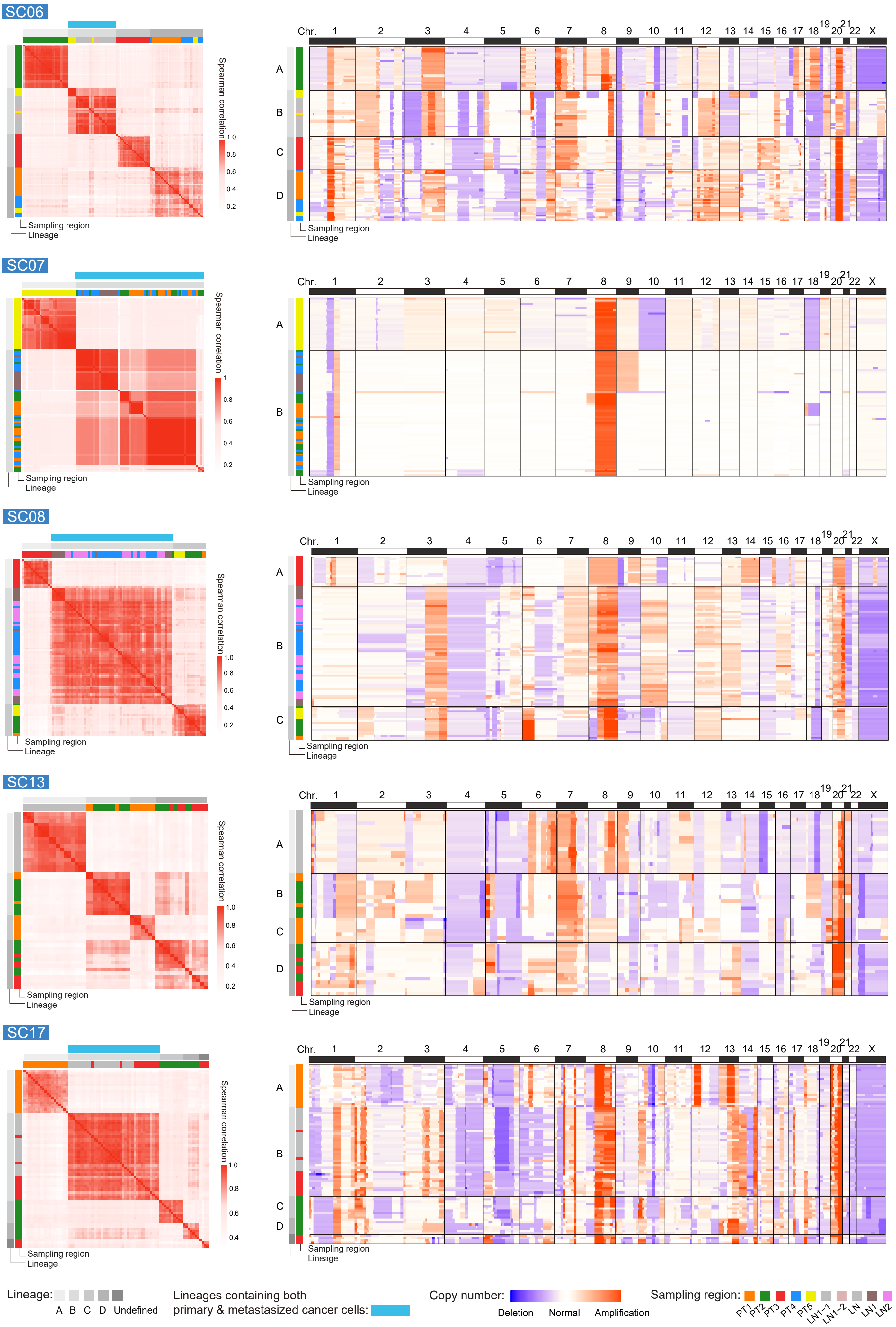
SC06
SC07
SC08
SC13
SC17
Spearman correlation
1.0
0.8
0.6
0.4
0.2
Sampling region
Lineage
Chr. 1 2 3 4 5 6 7 8 9 10 11 12 13 14 15 16 17 18 19 20 21 22 X
A
B
C
D
Lineage:
A B C D Undefined
Lineages containing both primary & metastasized cancer cells:
Copy number:
Deletion Normal Amplification
Sampling region:
PT1 PT2 PT3 PT4 PT5 LN1-1 LN1-2 LN LN1 LN2

Figure S5

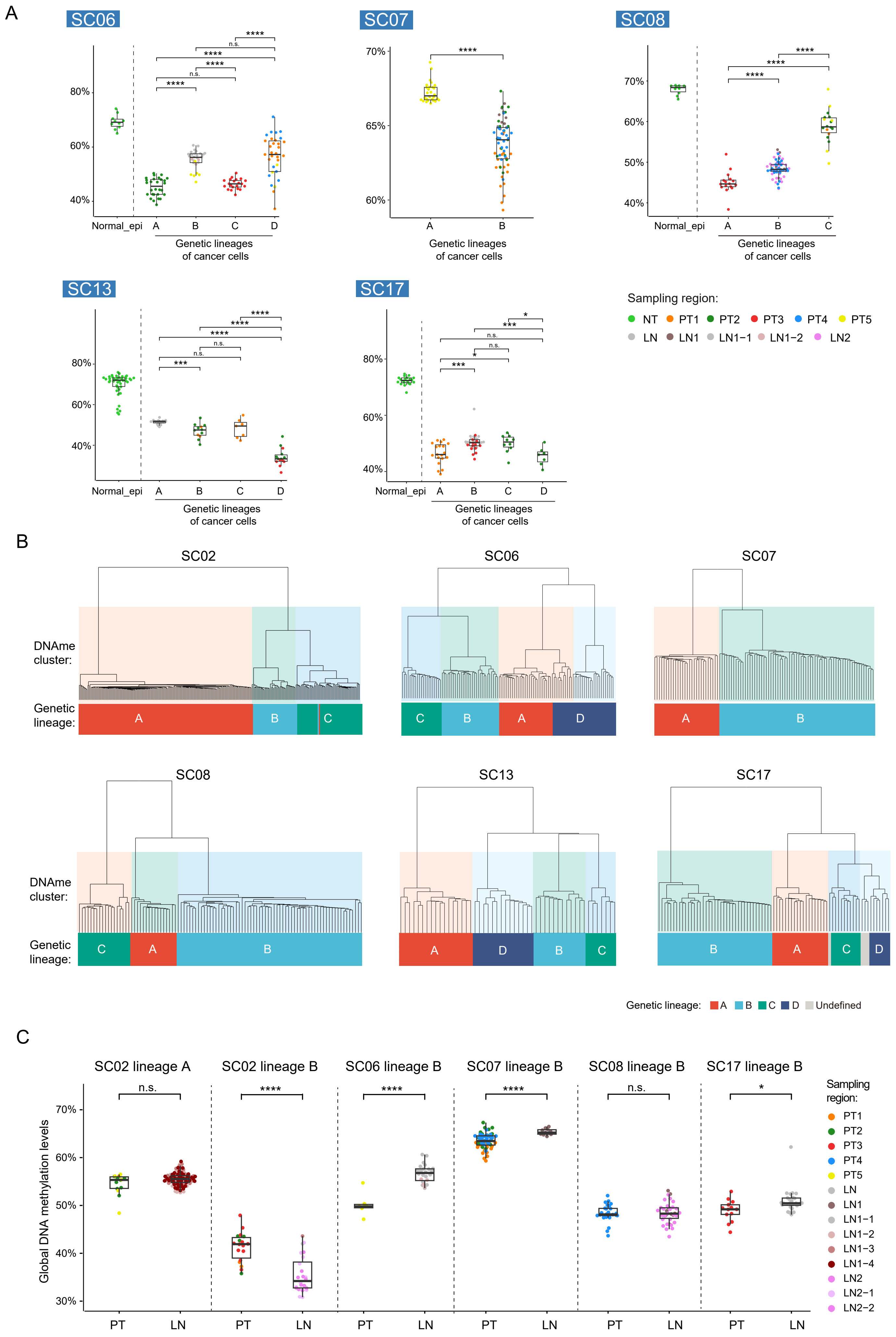

A
SC06
SC07
SC08
SC13
SC17
Normal_epi
Genetic lineages of cancer cells
Sampling region:
NT
PT1
PT2
PT3
PT4
PT5
LN
LN1
LN1−1
LN1−2
LN2
B
SC02
SC06
SC07
SC08
SC13
SC17
DNAme cluster:
Genetic lineage:
Genetic lineage: A B C D Undefined
C
SC02 lineage A
SC02 lineage B
SC06 lineage B
SC07 lineage B
SC08 lineage B
SC17 lineage B
Global DNA methylation levels
PT
LN
Sampling region:
PT1
PT2
PT3
PT4
PT5
LN
LN1
LN1−1
LN1−2
LN1−3
LN1−4
LN2
LN2−1
LN2−2

Figure S6

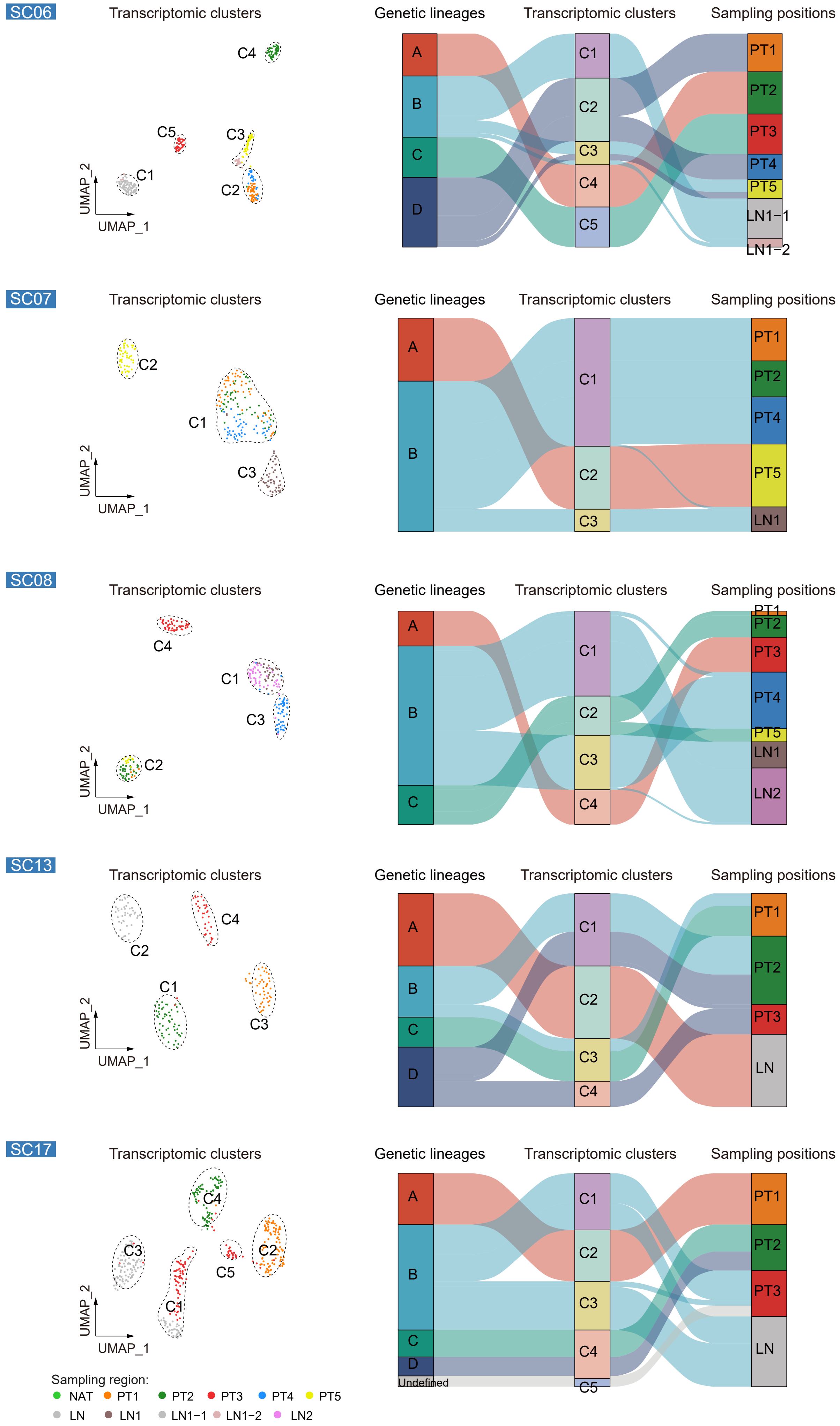
SC06
Transcriptomic clusters
C4
C5
C3
C1
C2
UMAP_2
UMAP_1
Genetic lineages
Transcriptomic clusters
Sampling positions
A
B
C
D
C1
C2
C3
C4
C5
PT1
PT2
PT3
PT4
PT5
LN1−1
LN1−2
SC07
Transcriptomic clusters
C2
C1
C3
UMAP_2
UMAP_1
Genetic lineages
Transcriptomic clusters
Sampling positions
A
B
C1
C2
C3
PT1
PT2
PT4
PT5
LN1
SC08
Transcriptomic clusters
C4
C1
C3
C2
UMAP_2
UMAP_1
Genetic lineages
Transcriptomic clusters
Sampling positions
A
B
C
C1
C2
C3
C4
PT1
PT2
PT3
PT4
PT5
LN1
LN2
SC13
Transcriptomic clusters
C2
C4
C1
C3
UMAP_2
UMAP_1
Genetic lineages
Transcriptomic clusters
Sampling positions
A
B
C
D
C1
C2
C3
C4
PT1
PT2
PT3
LN
SC17
Transcriptomic clusters
C4
C3
C2
C5
C1
UMAP_2
UMAP_1
Genetic lineages
Transcriptomic clusters
Sampling positions
A
B
C
D
Undefined
C1
C2
C3
C4
C5
PT1
PT2
PT3
LN
Sampling region:
NAT
PT1
PT2
PT3
PT4
PT5
LN
LN1
LN1−1
LN1−2
LN2

Figure S7

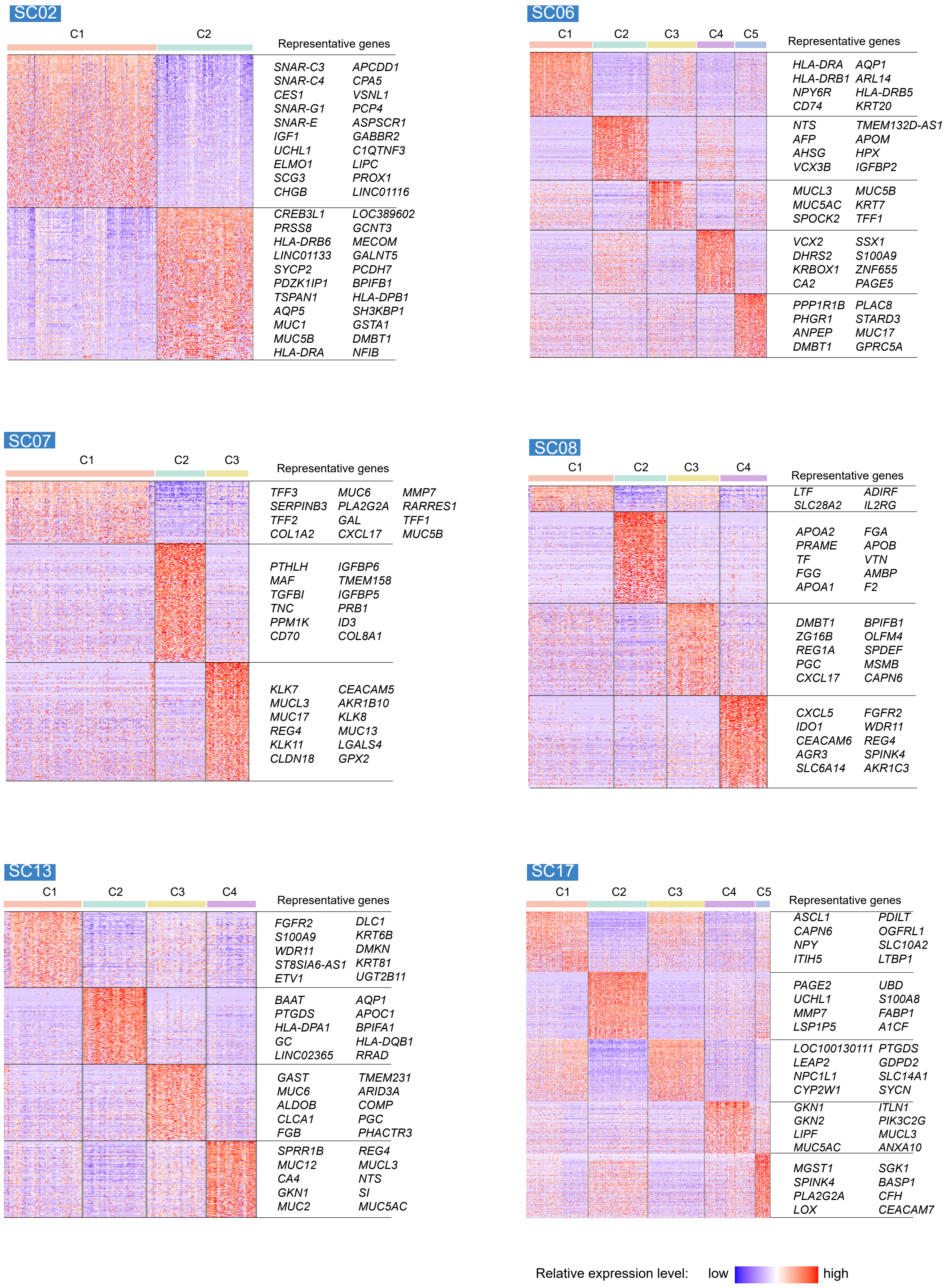
SC02
C1
C2
Representative genes
SNAR-C3 APCDD1
SNAR-C4 CPA5
CES1 VSNL1
SNAR-G1 PCP4
SNAR-E ASPSCR1
IGF1 GABBR2
UCHL1 C1QTNF3
ELMO1 LIPC
SCG3 PROX1
CHGB LINC01116
CREB3L1 LOC389602
PRSS8 GCNT3
HLA-DRB6 MECOM
LINC01133 GALNT5
SYCP2 PCDH7
PDZK1IP1 BPIFB1
TSPAN1 HLA-DPB1
AQP5 SH3KBP1
MUC1 GSTA1
MUC5B DMBT1
HLA-DRA NFIB
SC06
C1
C2
C3
C4
C5
Representative genes
HLA-DRA AQP1
HLA-DRB1 ARL14
NPY6R HLA-DRB5
CD74 KRT20
NTS TMEM132D-AS1
AFP APOM
AHSG HPX
VCX3B IGFBP2
MUCL3 MUC5B
MUC5AC KRT7
SPOCK2 TFF1
VCX2 SSX1
DHRS2 S100A9
KRBOX1 ZNF655
CA2 PAGE5
PPP1R1B PLAC8
PHGR1 STARD3
ANPEP MUC17
DMBT1 GPRC5A
SC07
C1
C2
C3
Representative genes
TFF3 MUC6 MMP7
SERPINB3 PLA2G2A RARRES1
TFF2 GAL TFF1
COL1A2 CXCL17 MUC5B
PTHLH IGFBP6
MAF TMEM158
TGFBI IGFBP5
TNC PRB1
PPM1K ID3
CD70 COL8A1
KLK7 CEACAM5
MUCL3 AKR1B10
MUC17 KLK8
REG4 MUC13
KLK11 LGALS4
CLDN18 GPX2
SC08
C1
C2
C3
C4
Representative genes
LTF ADIRF
SLC28A2 IL2RG
APOA2 FGA
PRAME APOB
TF VTN
FGG AMBP
APOA1 F2
DMBT1 BPIFB1
ZG16B OLFM4
REG1A SPDEF
PGC MSMB
CXCL17 CAPN6
CXCL5 FGFR2
IDO1 WDR11
CEACAM6 REG4
AGR3 SPINK4
SLC6A14 AKR1C3
SC13
C1
C2
C3
C4
Representative genes
FGFR2 DLC1
S100A9 KRT6B
WDR11 DMKN
ST8SIA6-AS1 KRT81
ETV1 UGT2B11
BAAT AQP1
PTGDS APOC1
HLA-DPA1 BPIFA1
GC HLA-DQB1
LINC02365 RRAD
GAST TMEM231
MUC6 ARID3A
ALDOB COMP
CLCA1 PGC
FGB PHACTR3
SPRR1B REG4
MUC12 MUCL3
CA4 NTS
GKN1 SI
MUC2 MUC5AC
SC17
C1
C2
C3
C4
C5
Representative genes
ASCL1 PDILT
CAPN6 OGFRL1
NPY SLC10A2
ITIH5 LTBP1
PAGE2 UBD
UCHL1 S100A8
MMP7 FABP1
LSP1P5 A1CF
LOC100130111 PTGDS
LEAP2 GDPD2
NPC1L1 SLC14A1
CYP2W1 SYCN
GKN1 ITLN1
GKN2 PIK3C2G
LIPF MUCL3
MUC5AC ANXA10
MGST1 SGK1
SPINK4 BASP1
PLA2G2A CFH
LOX CEACAM7
Relative expression level: low high

Figure S8

A

Top 20 enriched pathways using the up-regulated genes of cancer cells with poorer differentiation states

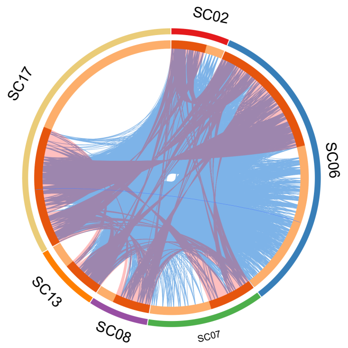

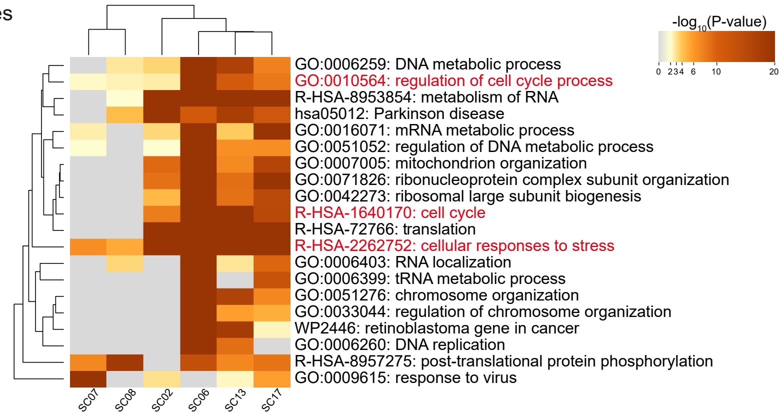

Top 20 enriched pathways using the up-regulated genes of cancer cells with better differentiation states

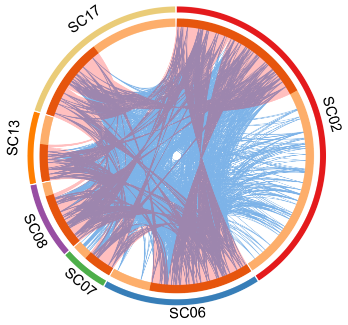

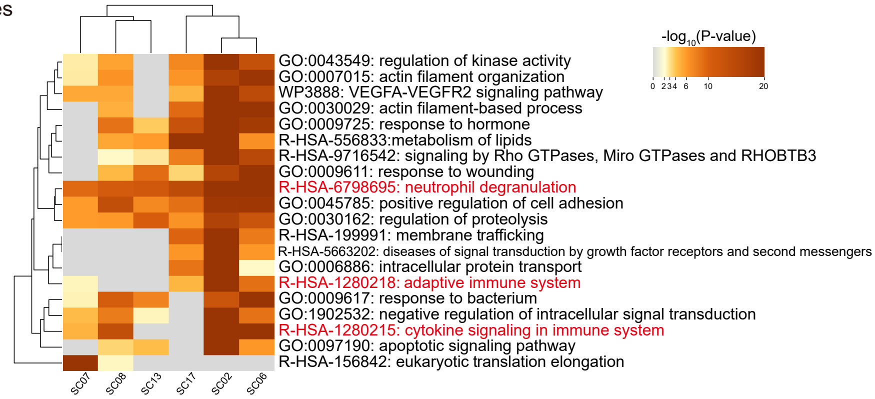

B

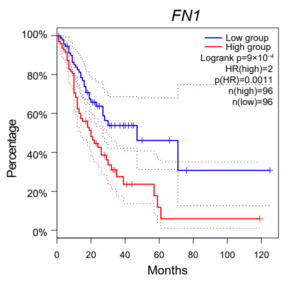

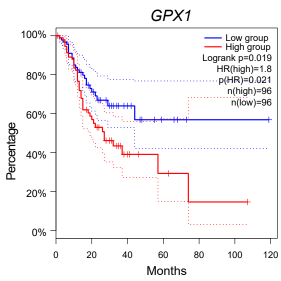

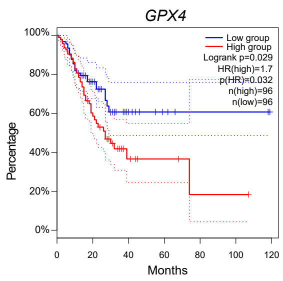

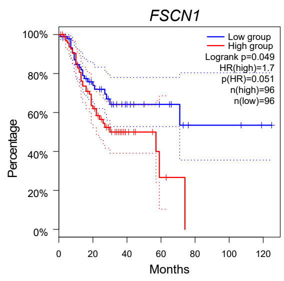

Figure S9

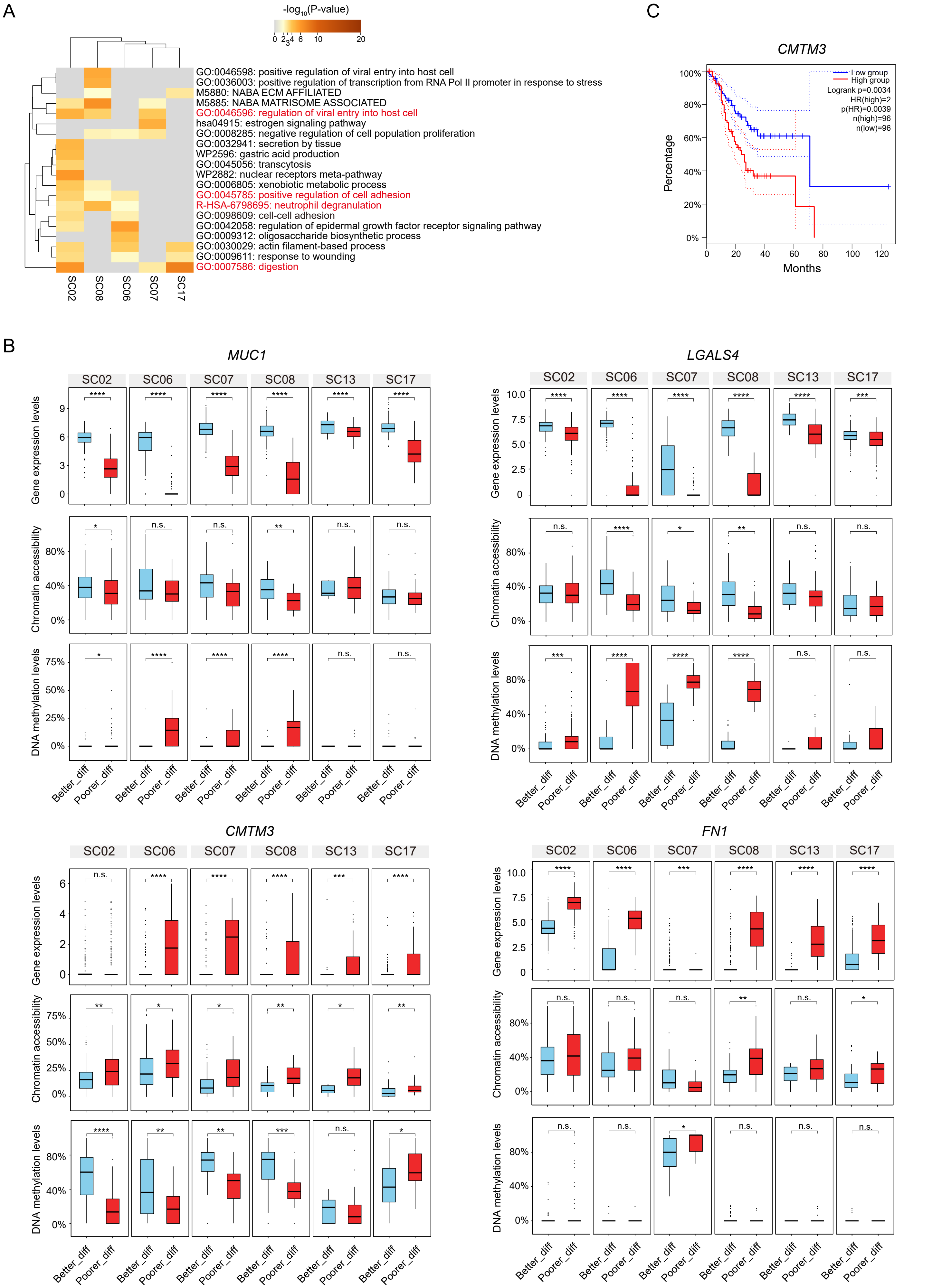

A
-log10(P-value)
0 2 3 4 6 10 20
GO:0046598: positive regulation of viral entry into host cell
GO:0036003: positive regulation of transcription from RNA Pol II promoter in response to stress
M5880: NABA ECM AFFILIATED
M5885: NABA MATRISOME ASSOCIATED
GO:0046596: regulation of viral entry into host cell
hsa04915: estrogen signaling pathway
GO:0008285: negative regulation of cell population proliferation
GO:0032941: secretion by tissue
WP2596: gastric acid production
GO:0045056: transcytosis
WP2882: nuclear receptors meta-pathway
GO:0006805: xenobiotic metabolic process
GO:0045785: positive regulation of cell adhesion
R-HSA-6798695: neutrophil degranulation
GO:0098609: cell-cell adhesion
GO:0042058: regulation of epidermal growth factor receptor signaling pathway
GO:0009312: oligosaccharide biosynthetic process
GO:0030029: actin filament-based process
GO:0009611: response to wounding
GO:0007586: digestion
SC02
SC08
SC06
SC07
SC17
C
CMTM3
Low group
High group
Logrank p=0.0034
HR(high)=2
p(HR)=0.0039
n(high)=96
n(low)=96
Percentage
Months
B
MUC1
LGALS4
CMTM3
FN1
SC02 SC06 SC07 SC08 SC13 SC17
Gene expression levels
Chromatin accessibility
DNA methylation levels
Better_diff
Poorer_diff
n.s.

Figure S10

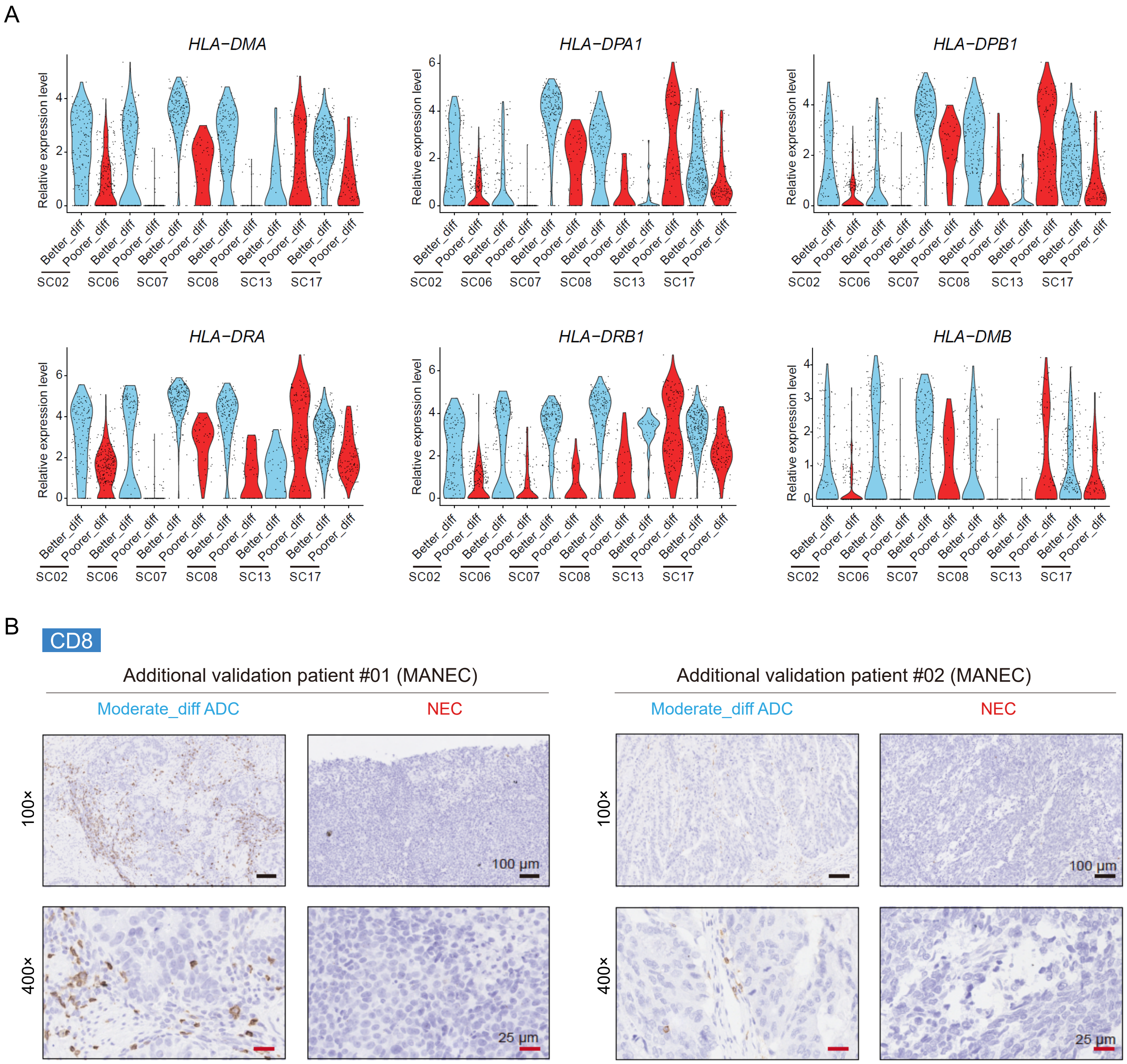
A
HLA−DMA
HLA−DPA1
HLA−DPB1
HLA−DRA
HLA−DRB1
HLA−DMB
Relative expression level
Better_diff
Poorer_diff
SC02
SC06
SC07
SC08
SC13
SC17
B
CD8
Additional validation patient #01 (MANEC)
Additional validation patient #02 (MANEC)
Moderate_diff ADC
NEC
100×
400×
100 μm
25 μm

Figure S1. Transcriptomic signatures of cancer cells compared with normal epithelial cells

(A) Clinical information of patients and multi-regional sampling strategy.

(B) The Venn plot showed overlapped gene number between normal_epi_colon highly expressed genes and up-regulated genes of GC cells.

(C) The dotplot showed the expression levels and expressed percentages of the 26 overlapped genes in (B).

(D) The dotplot showed the expression levels and expressed percentages of the 26 overlapped genes in (B) in each patient.

(E) The IHC staining results from Human Protein Atlas.

(F) The H&E staining showed intestinal metaplasia of SC14 and SC15 NAT.

(G) The Venn plot showed overlapped gene number between normal_epi_stomach highly expressed genes and down-regulated genes of GC cells.

(H) The gene expression levels, and DNA methylation levels and chromatin accessibility levels of related promoter regions of representative genes. The white diamonds represent the mean value of each group. Wilcoxon rank-sum test, ****P-value < 0.0001.

Figure S2. Abnormal DNA methylation levels of repeat elements in gastric cancer cells

(A) The global DNA methylation levels of all patients.

(B) The global DNA methylation levels of repeat elements.

(C) The global DNA methylation levels of L1.

(D) The IHC staining of L1 ORF1p protein showed abnormal expression in cancer cells. Scale bar, 100 μm.

Figure S3. Differentially methylated promoters between cancer cells and normal epithelial cells

(A) and (B) The multidimensional scaling (MDS) projection of the promoter DNA methylation levels (A) and chromatin accessibility levels (B).

(C) The heatmaps showed the DMPs between cancer cells and normal_epi_stomach. The normal_epi_stomach from all the patients are used as control. White represents missing values. Sam_pos, sampling position.

(D) The black dots indicate that the gene promoters are hypermethylated or hypomothylated in the patients significantly. The gene names and the number of patients by which the genes are shared were listed below.

Figure S4. Identification of genetic lineages of mGC patients

The left heatmaps showed the correlations of SCNA profiles. The right heatmaps showing the SCNA profiles of each genetic lineage of each patient. The cells are ordered according to the results of hierarchical clustering of SCNA profiles. The skyblue bar labeling the genetic lineages containing both primary cancer cells and metastasized cancer cells.

Figure S5. The relationships between genetic lineages and DNA methylation levels of cancer cells

(A) The global DNA methylation levels of each genetic lineage.

(B) The hierarchical clustering of genome-wide DNA methylation profiles (1-kb tiles).

(C) The global DNA methylation levels of cancer cells in PT and LN within each genetic lineage. Wilcoxon rank-sum test, ****P-value < 0.0001, ***P-value < 0.001, **P-value < 0.01, *P-value < 0.05. n.s., not significant.

Figure S6. The relationships between genetic lineages, transcriptomic clusters and sampling positions

The UMAP projection showed cancer cells of each mGC patient with transcriptomic cluster ID labelled. The alluvial plot showed the relationships between genetic lineages, transcriptomic clusters and sampling positions.

Figure S7. DEGs of each transcriptomic cluster for 6 mGC patients

The heatmaps showed the relative expression levels of top 200 DEGs of each transcriptomic cluster for 6 mGC patients. Representative genes of each clusters were showed.

Figure S8. The transcriptomic features of two major differentiation states within each mGC patient

(A) The enriched pathways of cancer cells with different differentiation states.

(B) The overall survival analysis of TCGA STAD dataset using the expression levels of some representative genes.

Figure S9. The genes with altered expression levels, promoter DNA methylation levels and chromatin accessibility shared by multiple mGC patients.

(A) The enriched pathway of the DEGs with altered DNA methylation and/or chromatin accessibility levels in their promoter regions.

(B) The gene expression levels, promoter chromatin accessibility levels and DNA methylation levels of cancer cells with different differentiation states. Wilcoxon rank-sum test, ****P-value < 0.0001, ***P-value < 0.001, **P-value < 0.01, *P-value < 0.05. n.s., not significant.

(C) The overall survival analysis of TCGA STAD dataset using the expression levels.

Figure S10. Distinct immune-related gene expression features of GC cells with different differentiation states.

(A) The gene expression levels of some MHC II related genes of 2 major differentiation states within each mGC patient.

(B) IHC staining of CD8 protein for two additional validation patients with MANEC.
